# Identification of key genes and pathways in scleral extracellular matrix remodeling in glaucoma: Potential therapeutic agents discovered using bioinformatics analysis

**DOI:** 10.7150/ijms.52846

**Published:** 2021-02-04

**Authors:** Di Hu, Junhong Jiang, Zhong Lin, Cong Zhang, Nived Moonasar, Shaohong Qian

**Affiliations:** 1 Department of Ophthalmology and Vision Science, Eye and ENT Hospital, Shanghai Medical College, Fudan University, Shanghai, China.; 2 The Eye Hospital, School of Ophthalmology and Optometry, Wenzhou Medical University, Wenzhou, Zhejiang, China.; 3 Caribbean Eye Institute, Valsayn, Trinidad and Tobago.

**Keywords:** glaucoma, sclera, extracellular matrix, text mining, drug discovery

## Abstract

**Background:** Glaucoma is a leading cause of irreversible blindness. Remodeling of the scleral extracellular matrix (ECM) plays an important role in the development of glaucoma. The aim of this study was to identify the key genes and pathways for the ECM remodeling of sclera in glaucoma by bioinformatics analysis and to explore potential therapeutic agents for glaucoma management.

**Methods:** Genes associated with glaucoma, sclera and ECM remodeling were detected using the text mining tool pubmed2ensembl, and assigned Gene Ontology (GO) biological process terms and Kyoto Encyclopedia of Genes and Genomes (KEGG) pathways using the GeneCodis program. A protein-protein interaction (PPI) network was constructed by STRING and visualized in Cytoscape, module analysis was performed using the Molecular Complex Detection (MCODE) plugin, and GO and KEGG analyses of the gene modules were performed using the Database of Annotation, Visualization and Integrated Discovery (DAVID) platform. The genes that clustered in the significant module were selected as core genes, and functions and pathways of the core genes were visualized using ClueGO and CluePedia. Lastly, the drug-gene interaction database was used to explore drug-gene interactions of the core genes to find drug candidates for glaucoma.

**Results:** We identified 125 genes common to “Glaucoma”, “Sclera”, and “ECM remodeling” by text mining. Gene functional enrichment analysis yielded 30 enriched GO terms and 20 associated KEGG pathways. A PPI network that included 60 nodes with 249 edges was constructed, and three gene modules were obtained using the MCODE. We selected 13 genes that clustered in module 1 as core candidate genes that were associated mainly with ECM degradation and cell proliferation and division. The HIF-1 signaling pathway, FOXO signaling pathway, PI3K-Akt signaling pathway and TGFB signaling pathway were found to be enriched. We found that 11 of the 13 selected genes could be targeted by 26 existing drugs.

**Conclusions:** The results showed that *VEGFA*, *TGFB1*, *TGFB2*, *TGFB3*, *IGF2*, *IGF1*, *EGF*, *FN1*, *KNG1*, *TIMP1*, *SERPINE1*, *THBS1*, and *VWF* were potential key genes involved to scleral ECM remodeling. Furthermore, 26 drugs were identified as potential therapeutic agents for glaucoma treatment and management.

## Introduction

Glaucoma is a progressive optic neuropathy characterized by progressive degeneration of retinal ganglion cells and optic nerve head (ONH) axons 1. The global prevalence of glaucoma is 3.54% in the population aged 40-80 years, and glaucoma was estimated to have affected 76 million people worldwide in 2020 2. Glaucoma is a leading cause of irreversible blindness, and it was estimated that 9.4 million people are bilaterally blind as a result of glaucoma worldwide 3. Glaucoma-related visual impairment can significantly reduce the quality of life, causing heavy economic burden to individuals and by extension to the society at large. With an aging global population, the number of patients with glaucoma is projected to grow to 111.8 million by 2040 2, and the overall burden of glaucoma treatment will continue to increase.

Currently, lowering of intraocular pressure (IOP) (by medication, laser/or filtering surgery) is the only available option to delay the development and progression of glaucoma 4. However, treatments that reduce IOP prevent vision loss in only 50%-70% of patients with glaucoma, and thus nearly a half of the patients will experience glaucomatous progression despite the IOP within the normal range 5, 6. Clearly, a more effective therapy for the treatment and prevention of glaucoma is urgently needed.

Retinal ganglion cell axons converge at the ONH, pass through the laminar cribrosa, enter the scleral canal and exit the eye 7. The biomechanical transmission of stress from IOP produces strain in the ONH and leads to retinal ganglion cell axonal injury and vascular perfusion deficiency, eventually leading to glaucomatous optic neuropathy 8, 9. ONH is the principal site of glaucomatous damage 10, and the IOP-related biomechanical response of ONH is determined by the mechanical properties of sclera, especially the adjacent peripapillary sclera 11.

Stiffening of the sclera leading to scleral rigidity is one of the primary structural changes in glaucoma 12, 13. Sclera consists of fibroblasts and extracellular matrix (ECM), which is made up of collagen, elastin, and proteoglycans 8, 14. Scleral fibroblasts are mechanosensitive, and elevated IOP can induce them to differentiate into myofibroblasts, resulting in ECM remodeling, which involves scleral fibroblast proliferation, followed by fibrosis of the sclera with alterations in the biomechanical properties 14, 15. Finite element modeling and ex vivo studies have shown that the biomechanical strain within the laminar cribrosa depends on the stiffness of the sclera, and that increased peripapillary sclera stiffness can reduce the laminar cribrosa strain 16, 17. In an *in vivo* experiment, mice with microfibril deficiency were shown to have increased susceptibility to glaucomatous damage 18, whereas increased mechanical stiffness of the sclera was found to result in increased glaucomatous damage in a mouse model 19. Therefore, scleral ECM remodeling might be a suitable drug target for preventing the biomechanical damage associated with glaucoma.

The discovery of new drug therapies using conventional approaches can be time-consuming and costly, whereas repurposing an existing drug to treat a condition beyond its original intent may be a more effective and less expensive alternative 20. Text mining of biomedical literature is as an effective method for hypothesis generation, because it can reveal novel associations between genes and pathologies 21. By combining text mining with biological knowledge and other analytical tools, new information about the potential to repurpose existing drugs can be obtained 22. The aim of this study was to investigate therapeutic targets and new drug therapies for glaucoma by mining the available published literature combined with biological databases and other analytical tools.

## Methods

### Text mining

Text mining was performed using pubmed2ensembl (http://pubmed2ensembl.ls.manchester.ac.uk/), a publicly available resource that links over 2,000,000 articles in PubMed to nearly 150,000 genes from 50 species in Ensembl 23. We used the search terms “Glaucoma”, “Sclera” and “ECM remodeling” from 100,000 relevant document IDs to produce a list of genes. “*Homo sapiens* (GRCh37)” was selected as the species dataset and “filter on MEDLINE: PubMed ID” was set to constrain the query result. The unduplicated genes were extracted and the intersection of gene hits from the three gene sets was retrieved as the text mining genes (TMGs).

### Biological process and pathway enrichment analysis of TMGs

We used GeneCodis (http://genecodis.cnb.csic.es/) to perform an enrichment analysis of the TMGs related to scleral ECM remodeling in glaucoma. GeneCodis is a web‑based server for functional analysis of gene lists that integrates different sources of information 24. The TMGs were used as the input set and analyzed using the gene ontology (GO) biological process categories, and genes with significantly enriched biological processes were selected and used for further analysis of enriched Kyoto Encyclopedia of Genes and Genomes (KEGG) pathway annotations. P =1.00E-07 was set as the cutoff. Genes involved in the significantly enriched KEGG pathways were used for further analysis.

### Integration of protein-protein interaction (PPI) network and module analysis

The Search Tool for the Retrieval of Interacting Genes (STRING, https://string-db.org/cgi/input.pl) is an online database resource that covers approximately 24.6 million proteins and more than 3.1 billion interactions originating from 5090 organisms 25. We used STRING (version 11.0) to construct PPI networks of the selected genes. The highest confidence score (0.900) was set as the minimum required interaction score. Molecular interaction networks were visualized using Cytoscape 26, and significant gene modules (clusters) from the PPI network were detected using the Molecular Complex Detection (MCODE) built in Cytoscape, with the following criteria: degree cutoff =2, node score cutoff=0.2, k-core=2, max depth=100 27.

### Gene ontology and KEGG pathway enrichment analysis of module genes

The GO functional and KEGG pathway enrichment analyses of significant module genes were performed using Database for Annotation, Visualization and Integrated Discovery (DAVID) (https://david.ncifcrf.gov/), an online tool for gene functional analysis 28. P < 0.05 was set as the cutoff. The GO (http://www.geneontology.org) database contains terms for the functional classification for genomic data under three main categories: biological processes, cellular component, and molecular function 29. KEGG (http://www.genome.ad.jp/kegg/) is a knowledge base for systematic analysis, annotation, and visualization of gene functions 30. The GO functional and KEGG pathway enrichment analyses of the core genes was performed and visualized using the Cytoscape plugins ClueGO (version 2.5.7), and CluePedia (version 1.5.7) 31. P < 0.01 was considered statistically significant.

### Drug-gene interactions

The drug-gene interaction database (DGIdb, www. dgidb.org) is a web resource that consolidates disparate data sources that describe drug-gene interactions and gene druggability 32. We used DGIdb (version 3.0) to explore drug-gene interactions in the significant module genes that were identidied as the potential targets for existing drugs or compounds.

## Results

### Identification of text mining genes (TMGs)

**Figure 1:**
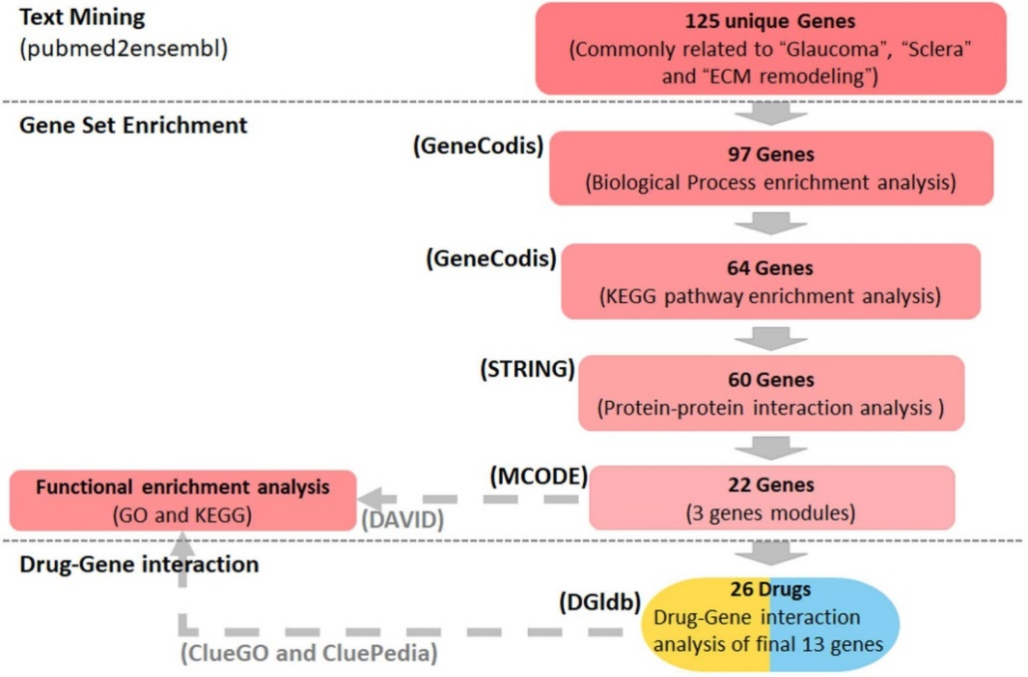
** Summary of the study design.** Text mining was conducted using pubmed2ensembl to identify genes associated with glaucoma, sclera, and extracellular matrix (ECM) remodeling. Gene set enrichment was performed using GeneCodis to detect genes enriched in gene ontology (GO) biological process terms and KEGG pathways. STRING and MCODE were used to construct a protein-protein interaction network and identify modules. The GO biological process terms and KEGG pathways were analyzed using DAVID and ClueGO. The drug list was obtained based on the gene-drug interaction analysis conducted using the drug-gene interaction database (DGIdb). KEGG: Kyoto Encyclopedia of Genes and Genomes.

**Figure 2:**
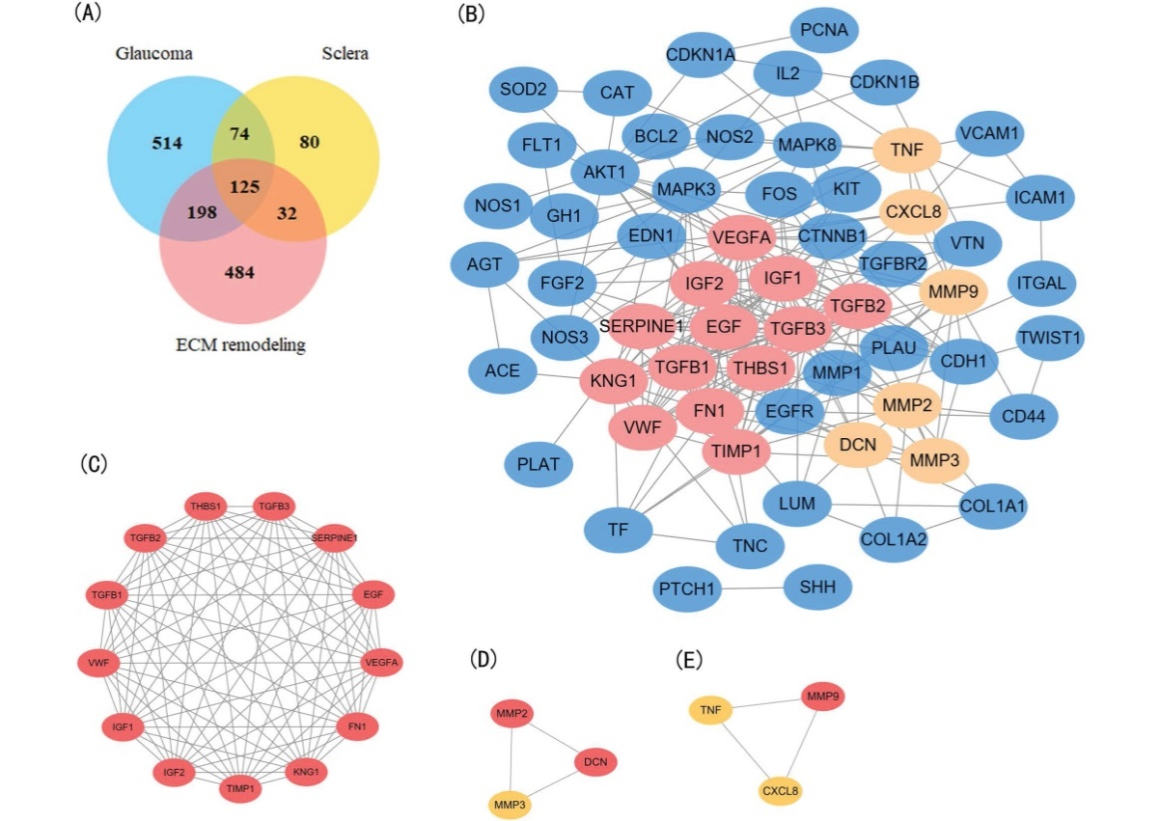
** Identification and enrichment analysis of the text mining genes (TMGs).** (A) Venn diagram of the TMGs related to glaucoma, extracellular matrix (EMC) remodeling, and sclera. The 125 genes that were common were considered to be related to scleral EMC remodeling. (B) The protein-protein interaction (PPI) network of the 64 target TMGs was visualized using Cytoscape. (C-E) The three modules were obtained from PPI network using MCODE. (C) Module 1, the most significant module with 13 nodes; (D) Module 2; (E) Module 3.

Using the text mining strategy described in the methods section (Figure 1), we obtain 911 unique genes related to glaucoma, 839 related to ECM remodeling, 311 related to sclera; among them, 125 genes were related to all three, and thus we considered them to be involved in scleral ECM remodeling in glaucoma (Figure 2A, Table S1).

### Enrichment analysis of TMGs

The GO biological process terms and KEGG pathway enrichment analyses using GeneCodis (with P=1.00E-07 as the cutoff) to identified the most enriched terms closely related to the pathology of scleral ECM remodeling in glaucoma. Thirty significantly enriched GO biological process annotations of 97 unique genes were identified. Among them, the five most enriched terms were “extracellular matrix organization” (P=3.47306E-27), “platelet degranulation” (P=2.44362E-19), “cytokine-mediated signaling pathway” (P=3.99419E-18), “response to drug” (P=3.82E-14), and “positive regulation of cell population proliferation” (P=1.45E-13), for 31, 20, 25, 22, and 27 TMGs, respectively (Table 1). Other highly enriched biological process terms included “response to hypoxia”, “wound healing”, “regulation of cell population proliferation”, “response to mechanical stimulus”, “extracellular matrix disassembly”, and “aging” (Table S2).

The KEGG pathway enrichment analysis identified 20 significant pathways that involved 64 TMGs. The five most significantly enriched pathways were “AGE-RAGE signaling pathway in diabetic complications” (P=1.04E-22), “Proteoglycans in cancer” (P=3.04E-19), “Pathways in cancer” (P=1.88327E-16), “HIF-1 signaling pathway” (P=1.17E-12), and “Relaxin signaling pathway” (P= 1.34E-12), involving 23, 26, 34, 16, and 17 TMGs, respectively (Table 2). Other highly enriched pathways included “PI3K-Akt signaling pathway”, “MAPK signaling pathway”, “Rheumatoid arthritis”, “FOXO signaling pathway”, “Focal adhesion” and “Endocrine resistance” (Table S3).

### PPI network construction, modular analysis, and identification of core candidate genes

A PPI network was constructed for the 64 target genes using STRING with confidence score >0.900. The network had a total of 60 nodes with 249 edges (Figure 2B). Among the 60 nodes, 22 hub node genes were identified with filtering node degree ≥10 (Table 3). Modular analysis was performed using the MCODE built in Cytoscape, and three modules were obtained (Figure 2C-E).

**Figure 3:**
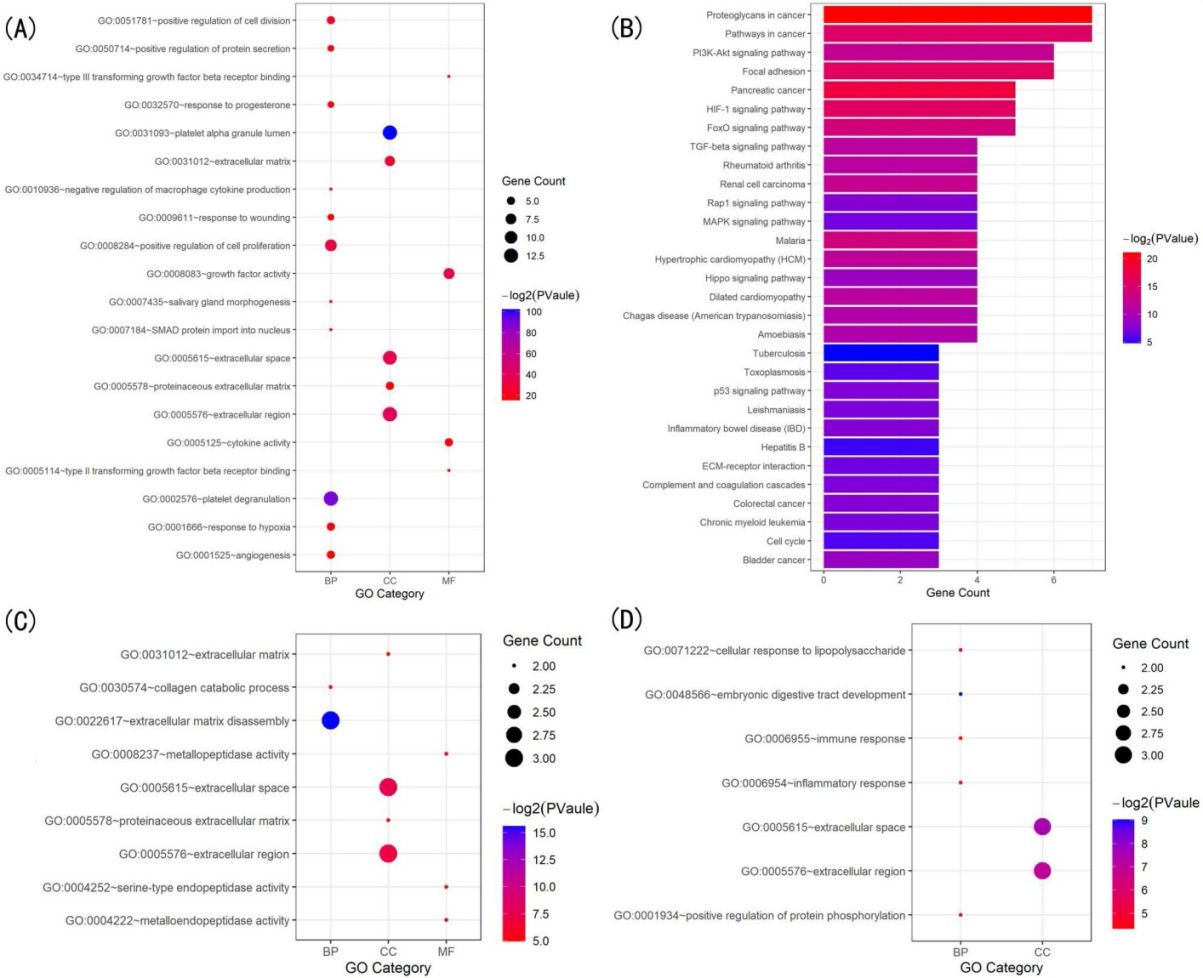
** Gene ontology (GO) and KEGG pathway analysis of the genes in the three modules.** (A) Top 20 significantly enriched GO terms in module 1. (B) Significantly enriched KEGG pathways in module 1. (C) Significantly enriched GO terms in module 2. (D) Significantly enriched GO terms in module 3. The functional and pathway enrichment analyses were performed using DAVID. KEGG: Kyoto Encyclopedia of Genes and Genomes.

The GO enrichment analysis showed that the three modules were related primarily to ECM degrading, cell proliferation and division which play crucial role in scleral ECM remodeling (Figure 3A,C,D). The pathway enrichment analysis showed that the genes in module 1 were associated with the HIF-1 signaling pathway, FOXO signaling pathway, PI3K-Akt signaling pathway and TGFB signaling pathway (Figure 3B). No specific KEGG pathways were associated with the genes in module 2, and the genes in module 3 were significantly associated with metallopeptidase or metalloendopeptidase activity, NOD-like receptor or RIG-I-like receptor signaling pathway, and NF-kappa B signaling pathway (Table 4).

**Figure 4:**
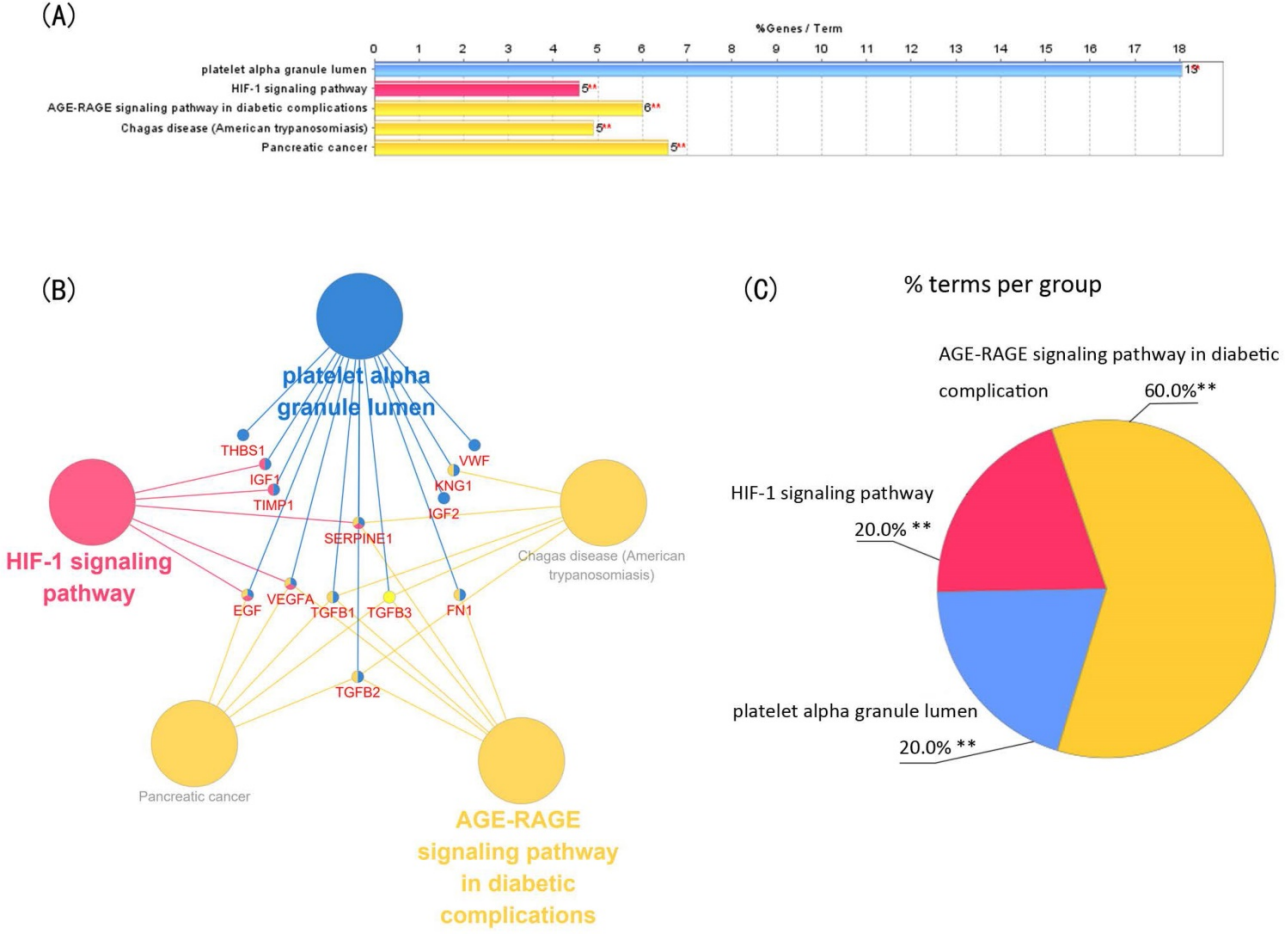
** Function analysis of the 13 core genes in module 1.** (A) Enriched gene ontology (GO) terms and KEGG pathways. (B) Functions and pathways of the core genes were visualized using ClueGO. (C) Distribution of the functions and pathways among the core genes. Each function or pathway is color coded. Corrected P <0.01 was considered statistically significant. KEGG: Kyoto Encyclopedia of Genes and Genomes.

Module 1 contained 13 genes with 88 edges, all of which were hub genes indicating that module 1 has a vital role in the PPI network. We selected the 13 hub genes, *TGFB1*, *TGFB2*, *TGFB3*, *VEGFA*, *IGF2*, *IGF1*, *EGF*, *FN1*, *KNG1*, *TIMP1*, *SERPINE1*, *THBS1* and *VWF*, as core candidate genes of the PPI network. The enrichment analysis indicated that these genes were significantly enriched in platelet alpha granule lumen, HIF-1 signaling pathway, and AGE-RAGE signaling pathway in diabetic complication (P <0.01, Figure 4).

### Drug-gene interaction analysis of core genes

**Figure 5:**
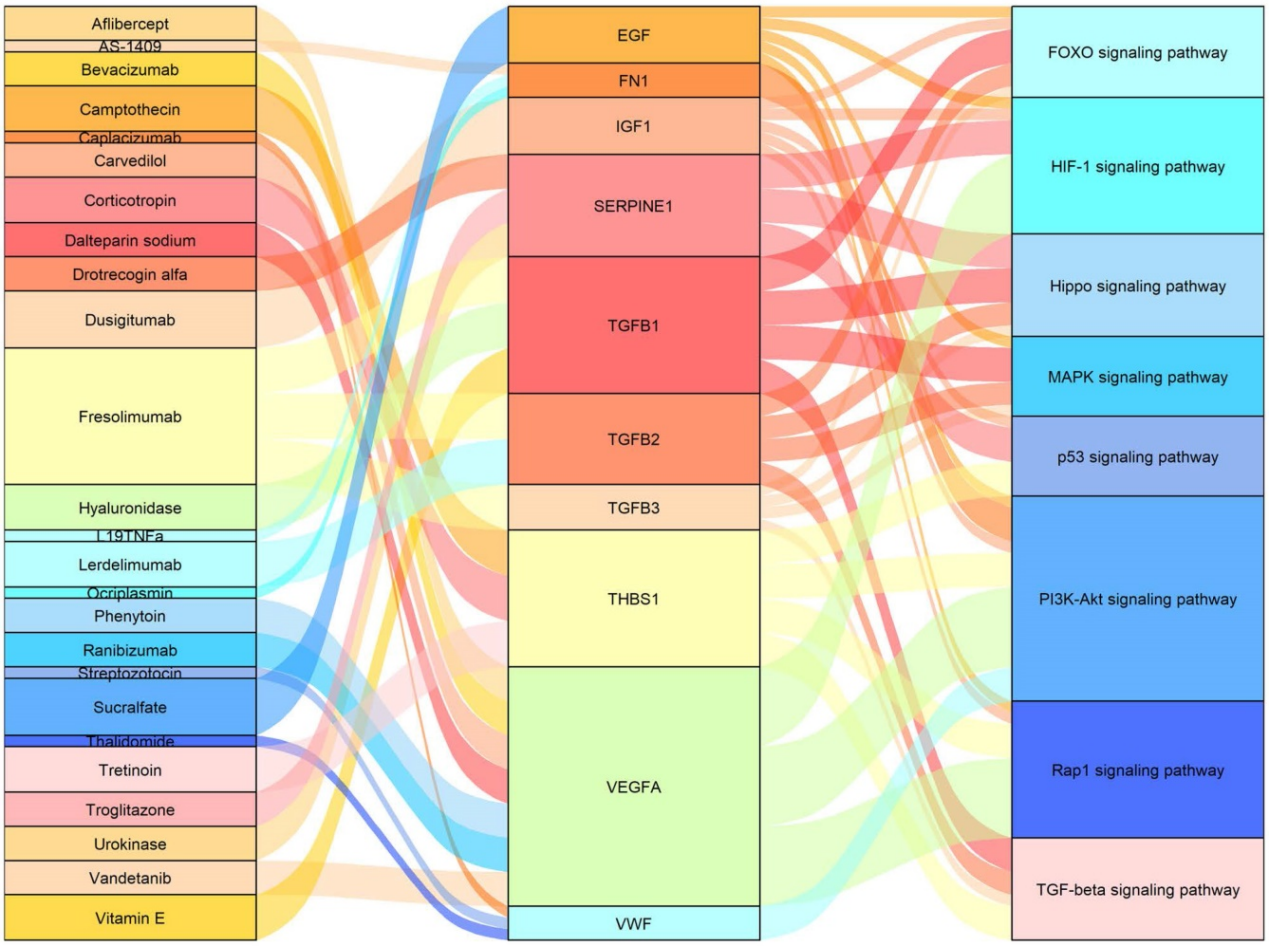
Sankey diagram showing the primary connections among drugs, genes, and pathways.

We used the 13 core genes as potential targets in a drug**-**gene interaction analysis, and obtained an initial list of 26 drugs (Table 5). Eleven of the 13 potential gene targets (the exceptions were *IGF2* and *TIMP1*) were predicted to be involved in drug-gene interactions. The primary connection among drugs, genes and pathways are shown in Figure 5.

## Discussion

Glaucoma is the leading cause of irreversible blindness globally. Reduction of IOP is the only proven method to treat glaucoma 7. However, many patients who are treated with IOP-lowering therapies have poor prognosis, and nearly a half of patients with glaucoma experience glaucomatous progression despite IOP reduction 33. Therefore, more therapeutic targets and prognostic biomarkers need to be identified. Stiffening of the sclera, particularly in peripapillary sclera, is one of the primary structural changes in glaucoma. It has been shown that elevated IOP can induce scleral fibroblasts to differentiate into myofibroblasts, resulting in ECM remodeling, followed by stiffening of the sclera 14, 15. The ONH is the primary site of damage in glaucoma, and stiffening of the peripapillary sclera significantly impacts the biomechanics of the ONH that eventually influence the susceptibility to glaucomatous damage 16-19. Therefore, ECM remodeling might be an attractive drug target for the prevention and treatment of glaucoma.

In the present study, we identified 125 genes that might be involved in scleral ECM remodeling associated with glaucoma using text mining strategy (Figure 1). The enriched GO biological process terms assigned to these genes were associated mainly with scleral ECM remodeling, including “positive regulation of cell population Proliferation”^34^, “response to hypoxia” 35, “aging” 36, “wound healing” 37, “response to mechanical stimulus” 38 and “positive regulation of MAP kinase activity” 39 (Table 1). The PPI network and enrichment analysis identified 13 core genes, *TGFB1*, *TGFB2*, *TGFB3*, *VEGFA*, *IGF2*, *IGF1*,* EGF*, *FN1*, *KNG1*, *TIMP1*, *SERPINE1*, *THBS1* and *VWF*, that were involved in the HIF-1 signaling pathway, TGFB signaling pathway, FOXO signaling pathway, and PI3K-Akt signaling pathway.

Transforming growth factor β (TGFB) belongs to a family of fibrogenic cytokine that includes TGFB1, TGFB2 and TGFB3 40. Inhibition of TGFB reduces α-SMA expression, myofibroblast differentiation, and proliferation of scleral fibroblasts in experimental glaucoma 41. Vascular endothelial growth factor A (VEGFA) is a central regulator of angiogenesis and vascular permeability, and is involved in angiogenesis, inflammatory cell migration, and fibroblast activity 42.VEGF-A can be induced by TGFB1 through the TGFB1-VEGF-A pathway, resulting in fibrosis in patients who undergo peritoneal dialysis 43. Inhibition of VEGF was shown to improve the surgical outcome of glaucoma surgery by reducing angiogenesis and fibrosis in experimental models 44. Insulin-like growth factor 1 (IGF1) is a pro‐fibrotic growth factor that stimulates fibroblast proliferation and collagen synthesis in idiopathic pulmonary fibrosis 45. IGF2 is involved in inflammation and fibrosis, and the IGF2 plasma levels were closely associated with the stages of liver fibrosis 46. Epidermal growth factor (EGF) is an essential growth factor in stimulating cell proliferation, which plays a role in the progression of fibrosis in the liver 47. Fibronectin 1(FN1) is involved in cell adhesion and migration, which is required for the accumulation of ECM components 48, and the interaction of FN1 with the cell surface is essential for TGFB-mediated activation of fibrogenic cells 49. SERPINE1, also called plasminogen activator inhibitor type 1 (PAI‐1), is a fibrinolysis inhibitor that contributes to fibrogenesis by decreasing the degradation of fibrin and other ECM proteins through the uPA/tPA/plasmin/MMP proteolytic system 50. Kininogen-1 (KNG1) is a multifunctional protein that plays an important role in fibrinolysis and inflammation 51. Tissue inhibitor of metalloproteinase 1 (TIMP1) is a glycoprotein in the TIMP family that plays a role in matrix remodeling by activating macrophages and inhibiting matrix metalloproteinases (MMPs) 52. Thrombospondin-1 (THBS1) belongs to the thrombospondin family of glycoproteins that are important components of the ECM 53. Gao et al. 54 demonstrated that *THBS1* expression was positively correlated with scleral ECM remodeling. Von Willebrand factor (VWF), a glycoprotein that functions to bridge platelets with an exposed collagen surface, is associated with the severity of organ fibrosis 55. These 13 core genes are the potential key genes that may be involved in scleral ECM remodeling.

The HIF-1 signaling pathway, one of the enriched KEGG pathways, has been shown to promote scleral myofibroblast transdifferentiation by regulating the actin cytoskeleton pathway, as well as ECM remodeling via the ECM receptor interaction pathway 35. HIF-1α was shown to enhance *VEGF-A* expression and promote fibrinogenesis via the HIF-1α-VEGF pathway in bronchiolitis obliterans 56. The TGFB pathway is acts as a primary signaling pathway in fibrogenesis by modulating the fibroblast phenotype and function, inducing myofibroblast transdifferentiation and α-SMA expression, and promoting matrix accumulation 57. TGFB-signaling and the abundances of TGFB1, TGFB2 and TGFB-activating protein were found to be elevated in the sclera of glaucomatous eyes versus the control sclera 41. Proteins in the FOXO family are growth factors and stress-regulated transcription factors that are involved in cellular differentiation, apoptosis, and cell proliferation. FOXO proteins can interact with TGFB-activated proteins to prevent fibrosis through β-catenin/FOXO1 signaling 58. The PI3K-Akt signaling pathway is an important regulatory pathway that is involved in the regulation of cell proliferation, migration, differentiation and angiogenesis 59. The PI3K-Akt signaling pathway also is involved in the pathological processes of fibrosis by regulating fibroblast migration and differentiation to myofibroblasts 60. Inhibition of the PI3K/AKT signaling pathway was shown to prevent scarring of the filtering bleb of glaucoma filtration surgery by inhibiting human conjunctival fibroblast migration, proliferation and ECM synthesis 61. These signaling pathways may play essential roles in the scleral stiffening associated with glaucoma.

Potential drugs identified by the drug-gene interaction search were classified as mainly TGFB inhibitors, VEGFA inhibitors, IGF inhibitors, fibrinolytic agents, antineoplastic agents, or immunomodulating agents. The TGFB inhibitors were mainly of hyaluronidase, lerdelimumab and fresolimumab. Hyaluronidases are enzymes that regulate the remodeling of ECM. Hyaluronidases have been shown to inhibit the growth of pulmonary fibrosis and decrease TGFB production and collagen deposition in mice 62. Lerdelimumab is a monoclonal immunoglobulin antibody that specifically and potently neutralizes human TGFB2. Lerdelimumab was found to reduce subconjunctival fibrosis by inhibiting TGFB2-induced Tenon's fibroblast migration and proliferation in *in vivo* animal experiments 63. Previous phase I and phase II clinical studies demonstrated that lerdelimumab reduced subconjunctival fibrosis in patients with primary glaucoma who had undergone trabeculectomy 64. Fresolimumab is a monoclonal antibody of TGFB that reduced myofibroblast infiltration and inhibited TGFB-regulated gene expression in systemic sclerosis 65.

More than a quarter of identified drugs target *VEGFA*, including ranibizumab, bevacizumab, aflibercept, carvedilol, dalteparin sodium, phenytoin and vandetanib. Ranibizumab, a monoclonal antibody fragment against VEGF-A, inhibits vascular permeability and has antiproliferative and antimetabolic effects on various cell lines. Several studies have indicated that ranibizumab may prevent scarring after glaucoma filtering surgery through the VEGF pathway 66. Bevacizumab is an IgG1 humanized monoclonal antibody that has been used to negate the effect of VEGF-A. Bevacizumab is approved by the US Food and Drug Administration. In clinical ophthalmology, bevacizumab has been used widely in the management of ophthalmic diseases, including choroidal neovascularization, diabetic macular edema and macular edema 67. Aflibercept is a human fusion protein of the IgG Fc region and VEGF-receptor VEGF-A that also has been used widely in the management of ophthalmic diseases 68. Carvedilol, is a nonselective-blocker that also has α1-adrenergic blocking, antioxidant and antiproliferative effects. Carvedilol was shown to attenuate liver cirrhosis by inhibiting angiogenesis through the VEGF-Src-ERK signaling pathway in human umbilical vascular endothelial cells 69. Dalteparin sodium is a low molecular weight heparin that is used widely in the treatment of thromboembolisms. In rat, dalteparin sodium minimized hepatic fibrogenesis caused by chronic carbon tetrachloride treatment 70. Vandetanib is an oral receptor tyrosine kinase inhibitor that potently inhibits VEGF receptor (VEGFR) tyrosine kinase activity. Vandetanib inhibits not only growth factor-induced phosphorylation of VEGFR and epidermal growth factor receptor (EGFR), but also mitogen-activated protein kinase (MAPK) and protein kinase B (Akt) 71. In mice, vandetanib reduced the degree of fibrosis on cutaneous wound healing 72.

Dusigitumab is a human antibody of IGF1 and IGF2. An *in vivo* experiment demonstrated that dusigitumab inhibited tumor growth of mouse embryonic fibroblast 73. Sucralfate is a complex salt of sucrose sulfate and aluminum hydroxide that induces significant increases in the expression of *EGF* and *TGFA* in gastric mucosal cells 74. Ocriplasmin, a recombinant protein with intrinsic action on collagen and fibronectin, is an effective nonsurgical treatment for vitreomacular traction. In rat, the intravitreal administration of microplasmin degraded fibronectin in the vitreoretinal interface and outer retina 75. Urokinase, also known as urokinase-type plasminogen activator (uPA), is a strong plasminogen activator that is involved in cell migration, wound healing and tissue remodeling. The action of uPA on podocytes was found to be associated with the decreased prevalence of tubulointerstitial fibrosis 76. Hydrochlorothiazide is a thiazide diuretic that is used in the treatment of hypertension and edema. In rat, hydrochlorothiazide was shown to reduce ischemic heart failure-induced fibrosis remodeling by inhibiting the angiotensin II signaling pathway 77. Tretinoin, a retinoid metabolite of naturally occurring vitamin A, stimulated the formation of new collagen, stimulated fibroblasts, prevented collagen loss, and inhibited the induction of metalloproteinases 78. Camptothecin is a DNA topoisomerase I inhibitor that blocks DNA synthesis and down-regulates *THBS1* expression in human thyroid carcinoma FTC-133 cells through the JNK/ATF-2 pathway 79. These drugs may have possible uses in the prevention of the scleral ECM remodeling in glaucoma. Further research is needed to confirm their possible new function.

Scleral ECM remodeling also is a key factor in the pathogenesis of myopia. In myopic human eyes, the sclera was found to be thinner, and the scleral ECM was decreased, compared with those in healthy eyes 80. The thinner sclera and the associated decrease in the synthesis of collagen, proteoglycan, and other scleral matrix components also have been observed in animal models of myopia 81-83. During myopia development, scleral ECM remodeling resulted in thinning of the sclera and reduced scleral resistance to IOP-related expansion which eventually contributed to excessive elongation of the ocular globe 84, 85. Scleral stiffening has been proposed as a therapy for myopia, and the 26 drugs identified in this study may also be potential therapeutic agents for the treatment of myopia.

In conclusion, we identified 13 hub genes, *VEGFA*, *TGFB1*, *TGFB2*, *TGFB3*, *IGF2*, *IGF1*, *EGF*, *FN1*, *KNG1*, *TIMP1*, *SERPINE1*, *THBS1* and *VWF*, that may be involved in the scleral ECM remodeling associated with glaucoma. These genes were enriched in the HIF-1 signaling pathway, FOXO signaling pathway, PI3K-Akt signaling pathway and TGFB signaling pathway. We also identified 26 potential drugs that may be help to guide future glaucoma therapies. The absence of experimental validation is a limitation of this study, and further experimental studies are required to verify the results.

## Supplementary Material

Supplementary tables.

## Figures and Tables

**Table 1:** Top 15 enriched gene ontology (GO) biological process terms assigned to the text mining genes

Process	Genes in query set	Total genes in genome	Corrected hypergeometric *P* value	Genes
Extracellular matrix organization	31	257	3.47E-27	*CDH1, CD44, VWF, VTN, VCAM1, TNF, THBS1, TGFBI, SPARC, BGN, SERPINE1, NID1, MMP14, MMP9, MMP3, MMP2, MMP1, MFAP2, LUM, ITGAL, ICAM1, TNC, FN1, FGF2, FBN1, ELN, COL18A1, DCN, VCAN, COL1A2, COL1A1.*
Platelet degranulation	20	125	2.44E-19	*CLU, MMRN1, VWF, VEGFA, TIMP1, THBS1, TGFB3, TGFB2, TGFB1, TF, SPARC, SOD1, SERPINE1, KNG1, IGF2, IGF1, APOA1, FN1, ALB, EGF.*
Cytokine-mediated signaling pathway	25	290	3.99E-18	*CDKN1A, CD4, VIM, VEGFA, VCAM1, TWIST1, TNF, TIMP1, TGFB1, BCL2, NOS2, MMP9, MMP3, MMP2, MMP1, KIT, IL8, IL2, ICAM1, FOS, FN1, FGF2, AKT1, F3, COL1A2.*
Response to drug	22	305	3.82E-14	*CDKN1B, CDKN1A, CDH1, CAT, TIMP2, THBS1, TGFBR2, TGFB2, SST, SOD1, BCL2, REN, PTCH1, APOA1, ICAM1, APEX1, FOS, ENG, EDN1, COL18A1, CTNNB1, COL1A1.*
Positive regulation of cell population Proliferation	27	557	1.45E-13	*CDKN1B, CNOT8, VEGFA, TIMP1, THBS1, TGFBR2, TGFB3, TGFB2, TGFB1, SHH, RPS4X, BCL2, KIT, IL2, IGF2, IGF1, TNC, FN1, FLT1, FGF2, AKT1, AGT, EGFR, EGF, EDN1, COL18A1, CTNNB1.*
Response to hypoxia	17	174	7.16E-13	*CDKN1B, CAT, VEGFA, THBS1, TGFBR2, TGFB3, TGFB2, BMP2, PLAU, PLAT, NOS2, NOS1, MMP14, MMP2, ICAM1, ENG, EDN1.*
Wound healing	14	103	1.99E-12	*TIMP1, TGFBR2, TGFB3, TGFB2, SPARC, IGF1, TNC, GSN, FN1, FGF2, ENG, EGFR, DCN, COL1A1.*
Regulation of cell population Proliferation	18	222	2.05E-12	*CLU, CDKN1B, TNF, TGFBR2, TGFB3, TGFB2, TGFB1, SPARC, SHH, PTCH1, PLAU, NOS2, KIT, TNC, ENG, AGT, EGFR, CTNNB1.*
Negative regulation of gene expression	20	310	4.16E-12	*CDKN1A, CD34, VEGFA, TNF, TGFB2, TGFB1, BMP2, SHH, SERPINF1, NOS2, MAX, IL8, IGF1, FGF2, AKT1, ENG, AGT, EDN1, ACE, CTNNB1.*
Positive regulation of gene expression	24	498	4.56E-12	*CLU, CD34, VIM, VEGFA, TWIST1, TNF, TGFB1, BMP2, SHH, MAPK8, MAPK3, NOS3, KIT, IL8, IGF1, TNC, GSN, GJA1, FN1, AKT1, F3, ENG, EGF, CTNNB1.*
Positive regulation of cell migration	18	255	1.66E-11	*VEGFA, THBS1, TGFB1, BMP2, SOD2, PLAU, MMP14, MMP9, KIT, IGF1, HSPA5, FLT1, F3, EGFR, EGF, EDN1, COL18A1, COL1A1.*
Aging	15	178	1.57E-10	*CAT, CALCA, TIMP2, TIMP1, TGFBR2, TGFB3, SOD1, MAPK3, SERPINF1, APEX1, GSN, FOS, AKT1, AGT, DCN.*
Cellular protein metabolic process	16	226	3.55E-10	*CALCA, TIMP1, TGFBI, TF, MMP2, MMP1, KNG1, IGF2, IGF1, APOA1, TNC, GSN, FN1, ALB, FBN1, VCAN.*
Regulation of blood pressure	10	63	1.50E-09	*CD34, CALCA, SOD2, SOD1, REN, NOS3, AGT, EDN1, ACE, COL1A2.*
Positive regulation of MAP kinase activity	10	63	1.50E-09	*VEGFA, TNF, TGFB1, KIT, GH1, FLT1, FGF2, EGFR, EGF, EDN1.*

**Table 2:** Top 10 enriched Kyoto Encyclopedia of Genes and Genomes (KEGG) pathways assigned to the text mining genes

Process	Genes in query set	Total genes in genome	Corrected hypergeometric *P* value	Genes
AGE-RAGE signaling pathway in diabetic complications	23	100	1.04E-22	*CDKN1B, VEGFA, VCAM1, TNF, TGFBR2, TGFB3, TGFB2, TGFB1, BCL2, MAPK8, MAPK3, SERPINE1, NOS3, MMP2, IL8, ICAM1, FN1, AKT1, F3, AGT, EDN1, COL1A2, COL1A1.*
Proteoglycans in cancer	26	205	3.04E-19	*CDKN1A, CD44, VTN, VEGFA, TWIST1, TNF, THBS1, TGFB2, TGFB1, SHH, PTCH1, MAPK3, PLAU, MMP9, MMP2, LUM, IGF2, IGF1, FN1, FGF2, AKT1, EGFR, DCN, CTNNB1, COL1A2, COL1A1.*
Pathways in cancer	34	531	1.88E-16	*CDKN1B, CDKN1A, CDH1, VEGFA, TGFBR2, TGFB3, TGFB2, TGFB1, BMP2, SHH, BCL2, PTCH1, MAPK8, MAPK3, NOS2, MMP9, MMP2, MMP1, MAX, KNG1, KIT, IL8, IL2, IGF2, IGF1, FOS, FN1, FGF2, AKT1, AGT, EGFR, EGF, EDN1, CTNNB1.*
HIF-1 signaling pathway	16	109	1.17E-12	*CDKN1B, CDKN1A, VEGFA, TIMP1, TF, BCL2, MAPK3, SERPINE1, NOS3, NOS2, IGF1, FLT1, AKT1, EGFR, EGF, EDN1.*
Relaxin signaling pathway	17	129	1.34E-12	*VEGFA, TGFBR2, TGFB1, MAPK8, MAPK3, NOS3, NOS2, NOS1, MMP9, MMP2, MMP1, FOS, AKT1, EGFR, EDN1, COL1A2, COL1A1.*
Chagas disease (American trypanosomiasis)	15	102	6.57E-12	*TNF, TGFBR2, TGFB3, TGFB2, TGFB1, MAPK8, MAPK3, SERPINE1, NOS2, KNG1, IL8, IL2, FOS, AKT1, ACE.*
Bladder cancer	11	41	1.05E-11	*CDKN1A, CDH1, VEGFA, THBS1, MAPK3, MMP9, MMP2, MMP1, IL8, EGFR, EGF.*
PI3K-Akt signaling pathway	23	354	5.42E-11	*CDKN1B, CDKN1A, VWF, VTN, VEGFA, THBS1, BCL2, MAPK3, NOS3, KIT, IL2, IGF2, IGF1, TNC, GH1, FN1, FLT1, FGF2, AKT1, EGFR, EGF, COL1A2, COL1A1.*
Colorectal cancer	13	86	1.41E-10	*CDKN1A, TGFBR2, TGFB3, TGFB2, TGFB1, BCL2, MAPK8, MAPK3, FOS, AKT1, EGFR, EGF, CTNNB1.*
Prostate cancer	13	97	6.18E-10	*CDKN1B, CDKN1A, BCL2, MAPK3, PLAU, PLAT, MMP9, MMP3, IGF1, AKT1, EGFR, EGF, CTNNB1.*

**Table 3:** Hub node genes in the protein-protein interaction network identified with filtering node degree ≥10

Number	Genes	Degree	MCODE Cluster	MCODE Node Status
1	*VEGFA*	24	Module 1	Seed
2	*IGF1*	21	Module 1	Clustered
3	*EGF*	20	Module 1	Clustered
4	*FN1*	20	Module 1	Clustered
5	*TGFB1*	19	Module 1	Clustered
6	*KNG1*	19	Module 1	Clustered
7	*TIMP1*	18	Module 1	Clustered
8	*TGFB3*	17	Module 1	Clustered
9	*AKT1*	16	--	Unclustered
10	*TGFB2*	16	Module 1	Clustered
11	*IGF2*	16	Module 1	Clustered
12	*SERPINE1*	15	Module 1	Clustered
13	*THBS1*	15	Module 1	Clustered
14	*MAPK3*	15	--	Unclustered
15	*EGFR*	14	--	Unclustered
16	*CTNNB1*	13	--	Unclustered
17	*MMP2*	13	Module 2	Clustered
18	*VWF*	13	Module 1	Clustered
19	*MMP9*	12	Module 3	Clustered
20	*DCN*	11	Module 2	Seed
21	*CDH1*	10	--	Unclustered
22	*EDN1*	10	--	Unclustered

**Table 4:** Kyoto Encyclopedia of Genes and Genomes (KEGG) pathway enrichment analysis of the genes in module 3

Term	Description	Count	*P* value
hsa05161	Hepatitis B	3	4.41E-04
hsa05219	Bladder cancer	2	0.011886
hsa05144	Malaria	2	0.014197
hsa05134	Legionellosis	2	0.015639
hsa04621	NOD-like receptor signaling pathway	2	0.016216
hsa04622	RIG-I-like receptor signaling pathway	2	0.02025
hsa05133	Pertussis	2	0.021688
hsa04064	NF-kappa B signaling pathway	2	0.025136
hsa05323	Rheumatoid arthritis	2	0.025423
hsa05142	Chagas disease	2	0.030011
hsa04620	Toll-like receptor signaling pathway	2	0.030583
hsa05146	Amoebiasis	2	0.030583
hsa04668	TNF signaling pathway	2	0.030869
hsa05160	Hepatitis C	2	0.038297
hsa04932	Non-alcoholic fatty liver disease (NAFLD)	2	0.043423
hsa05202	Transcriptional misregulation in cancer	2	0.047968
hsa05164	Influenza A	2	0.049953

**Table 5:** Details of the 26 drugs that potentially target 11 of the 13 core genes

Number	Drug	Genes	Interaction	Score	Drug Class	Approved?	Reference (PubMed ID)
1	Ranibizumab**	*VEGFA*	inhibitor	14	antineoplastic agents, ocular vascular disorder agents	Yes*	18046235
2	Bevacizumab	*VEGFA*	inhibitor	10	antineoplastic agent, immunomodulating agents	Yes*	15705858
3	Aflibercept**	*VEGFA*	inhibitor	7	antineoplastic agent, ocular vascular disorder agents	Yes*	22813448
4	Carvedilol	*VEGFA*	other	5	vasodilator agents, neurotransmitter agents	Yes*	15071347
5	Dalteparin sodium	*VEGFA*	inhibitor	4	hematologic agents, fibrinolytic agents	Yes	21091776
6	Phenytoin	*VEGFA*	N/A	2	anticonvulsants	Yes*	15458527
7	Vandetanib	*VEGFA*	inhibitor	1	antineoplastic and immunomodulating agent	Yes*	None found
8	Dusigitumab	*IGF1*	inhibitor	1	not available	No	None found
9	Sucralfate	*EGF*	inducer	4	antiulcer agent, antimuscarinics	Yes*	8578218
10	Ocriplasmin**	*FN1*	cleavage	3	not available	Yes	23193358
11	AS-1409	*FN1*	binder	2	antineoplastic agent	No	None found
12	L19TNFa	*FN1*	N/A	1	fibronectin binding agent	No	None found
13	Urokinase	*SERPINE1*	N/A	7	thrombolytic agents, fibrinolytic agents	Yes*	12709915
14	Troglitazone	*SERPINE1*	antagonist	6	hypoglycemic agents	No	10871202
15	Drotrecogin alfa	*SERPINE1*	N/A	5	antithrombotic agents, fibrinolytic agents,	Yes*	12004248
16	Hydrochlorothiazide	*KNG1*	inhibitor	1	antihypertensive agents, hypotensive agents	Yes*	None found
17	Tretinoin	*THBS1*	N/A	3	antineoplastic agents, dermatologic agents, immunosuppressive agents	Yes*	9447832
18	Corticotropin	*THBS1*	N/A	2	immunosuppressive agents	Yes*	8698834
19	Camptothecin	*THBS1*	N/A	2	antineoplastic agents	Yes*	16962673
20	Caplacizumab	*VWF*	inhibitor	3	antithrombotic	No	None found
21	Streptozotocin	*VWF*	inhibitor	3	antibiotics, antineoplastic agents, immunosuppressive agents	Yes*	16422885
22	Thalidomide	*VWF*	N/A	2	antineoplastic agents	Yes*	12871448
23	Hyaluronidase	*TGFB1*	inhibitor	5	not available	Yes*	9435505
24	Vitamin E	*TGFB1*	N/A	3	antioxidants	Yes*	1505665
25	Lerdelimumab	*TGFB2*	inhibitor	2	monoclonal antibody	No	None found
26	Fresolimumab	*TGFB1, TGFB2, TGFB3*	inhibitor	2	antineoplastic agents	No	None found

**Drugs that have been used to treat ophthalmic disease. *Drugs that have been approved by the US Food and Drug Administration.
