# Supplementary material for: Identification of key genes and pathways in scleral extracellular matrix remodeling in glaucoma: Potential therapeutic agents discovered using bioinformatics analysis
Source: Int J Med Sci. 2021 Feb 4;18(7):1554–65. doi: 10.7150/ijms.52846 (PMC7976561; doi:10.7150/ijms.52846)
Supplement: Supplementary file 1 — Supplementary tables. [file ijmsv18p1554s1.pdf]

Table S1. Detailed gene lists related with Glaucoma, Sclera and Extracellular matrix (ECM) remodeling from text mining.

Glaucoma related genes	Sclera related genes	ECM remodeling related genes	Common genes
GSTM1	*NID1*	*AKT3*	*NID1*
TAP2	*EPRS*	*NID1*	*TGFB2*
LGALS8	*TGFB2*	*ACTA1*	*HSD11B1*
NID1	*PROX1*	*PARP1*	*CD34*
TBCE	*IRF6*	*MIXL1*	*REN*
MIXL1	*HSD11B1*	*TGFB2*	*DES*
LBR	*PLXNA2*	*PROX1*	*MPZ*
EPRS	*CD34*	*HSD11B1*	*ABCB6*
TGFB2	*CD46*	*CD34*	*FN1*
IRF6	*CR2*	*IL10*	*RPE*
HSD11B1	*CD55*	*REN*	*SST*
PLXNA2	*REN*	*FMOD*	*KNG1*
CD34	*FMOD*	*CHI3L1*	*VCAM1*
CD46	*PRELP*	*CHIT1*	*F3*
IL10	*ADORA1*	*AGXT*	*SOD2*
NFASC	*CFH*	*GPC1*	*RHO*
REN	*PRG4*	*PTPRC*	*SHH*
OPTC	*MYOC*	*COL6A3*	*NOS3*
CHI3L1	*SELE*	*PTGS2*	*GJA1*
RNPEP	*SLC4A3*	*LAMC2*	*TIMP2*
LMOD1	*DES*	*SOAT1*	*MKI67*
PER2	*MPZ*	*FASLG*	*APOA1*

PTPRC	*ABCB6*	*SERPINE2*	*CALD1*
PRLH	*USF1*	*SELP*	*C2*
CRB1	*CD244*	*POU2F1*	*VAMP8*
CFH	*VANGL2*	*DES*	*MMP3*
PTGS2	*FN1*	*DDR2*	*MMP1*
PDC	*NES*	*MPZ*	*GH1*
C1orf27	*RPE*	*ABCB6*	*SLC17A5*
DHX9	*TFPI*	*ADAMTS4*	*NOVA2*
LAMC1	*NBPF14*	*IHH*	*DMPK*
SOAT1	*SST*	*CYP27A1*	*NOS1*
PSMD1	*OLFML3*	*NHLH1*	*ACE*
PRDX6	*KNG1*	*CRP*	*PVR*
TNFSF4	*CRYGS*	*IGFBP5*	*SERPINE1*
FASLG	*ADORA3*	*NTRK1*	*ACHE*
MYOC	*GCG*	*FN1*	*ENG*
COL4A3	*VCAM1*	*NES*	*BGN*
SELE	*F3*	*RPE*	*CD79A*
SELL	*MBNL1*	*CREB1*	*GALNS*
F5	*PLG*	*MUC1*	*TGFB1*
SELP	*SOD2*	*NRP2*	*VEGFA*
ATP1B1	*RHO*	*SHC1*	*HSPA5*
DPT	*VIPR2*	*BMPR2*	*COL1A1*
SLC4A3	*B3GAT1*	*SUMO1*	*IGF1*
DES	*NLRP5*	*CASP8*	*GSN*
PBX1	*SHH*	*S100A1*	*COL1A2*
MPZ	*LIMS1*	*S100A4*	*DCN*

ABCB6	*NOS3*	*FLG*	*LUM*
USF1	*HGS*	*CTSK*	*TNC*
CD244	*HGD*	*CTSS*	*GFAP*
VANGL2	*GJA1*	*ECM1*	*CDH1*
PEX19	*TIMP2*	*TFPI*	*CDKN1A*
ATP1A2	*MKI67*	*CALCRL*	*PLAU*
PNKD	*KLK1*	*ITGAV*	*MFAP2*
CRP	*APOA1*	*MFI2*	*CTNNB1*
DARC	*TFG*	*TTN*	*C10orf27*
IGFBP2	*CALD1*	*ATF2*	*ELN*
NTRK1	*C2*	*NGF*	*PTCH1*
FN1	*PAEP*	*TP63*	*MMP2*
LMNA	*TSPAN33*	*SST*	*CNOT8*
RPE	*CNTN3*	*ITGA6*	*EDN1*
MAP2	*DBH*	*ADIPOQ*	*ICAM1*
CREB1	*VAMP8*	*KNG1*	*VIM*
MUC1	*MITF*	*CRYGS*	*AGT*
BMPR2	*MTF1*	*GAD1*	*TWIST1*
NPR1	*MMP3*	*THPO*	*TIMP1*
CFLAR	*MMP1*	*CSF1*	*MMRN1*
HSPD1	*HPD*	*FAP*	*WAS*
STAT1	*FLNB*	*DPP4*	*FOS*
MTMR11	*GH1*	*PIK3CA*	*CYCS*
PHGDH	*TYR*	*TNFSF10*	*CAT*
APOD	*SLC17A5*	*CLDN11*	*TGFB3*
OPA1	*NOVA2*	*COL11A1*	*CD44*

NGF	*SIX5*	*EXTL2*	*NOS2*
SST	*DMPK*	*VCAM1*	*VTN*
OLFML3	*NOS1*	*SERPINI1*	*SMTN*
KNG1	*G6PD*	*DPYD*	*EGF*
CAPZA1	*ACE*	*F3*	*TGFBI*
CHRD	*RIMS1*	*TNFAIP6*	*KRT7*
CLCN2	*SFRS16*	*CTSZ*	*IL2*
PSMD2	*PVR*	*CXCR4*	*FGF2*
ABCC5	*PLAUR*	*CYR61*	*NUDT6*
PARL	*HCFC1*	*THBS2*	*MAPK3*
GSTM3	*MATN1*	*MGAT5*	*TNF*
SORT1	*HVCN1*	*CPB1*	*ITGAL*
MFN1	*TRIM37*	*AGTR1*	*MAPK8*
GPSM2	*SERPINE1*	*LPHN2*	*TGFBR2*
TANK	*ACHE*	*CFC1*	*TF*
TNFSF10	*COIL*	*PLG*	*GLB1*
CNTF	*ENG*	*LPA*	*EGFR*
7-Mar	*BGN*	*IGF2R*	*RPS4X*
COL11A1	*CD79A*	*SOD2*	*MMP9*
OLFM3	*RPS19P3*	*VIL2*	*MIP*
VCAM1	*PELI1*	*MAP3K2*	*SPARC*
BCHE	*MUT*	*RPE65*	*AKT1*
DPYD	*GALNS*	*GLI2*	*VCAN*
F3	*CETN2*	*LEPR*	*MAX*
ABCA4	*TGFB1*	*ESR1*	*CALCA*
P2RY1	*DHCR7*	*ATP2C1*	*IL8*

LCT	*GAL*	*IL1RN*	*FBN1*
DLL1	*VEGFA*	*IL1B*	*ALB*
CYR61	*CHAD*	*IL1A*	*COL18A1*
CP	*HSPA5*	*RHO*	*CTSD*
AGTR1	*AMT*	*JUN*	*PLAT*
LPHN2	*COL1A1*	*GRM1*	*BMP2*
PARK2	*IGF1*	*RAB7A*	*KIT*
PCOLCE2	*HHEX*	*SHH*	*PCNA*
ATP1B3	*OCM2*	*LIMS1*	*CDKN1B*
ZBTB38	*TAC1*	*ST14*	*GP1BA*
COPB2	*GSN*	*IL11*	*MBP*
SOD2	*SLC25A13*	*LRP8*	*BCL2*
PTGER3	*COL1A2*	*TNFAIP3*	*IGF2*
TMEM22	*ODZ1*	*FLI1*	*CLU*
EPHB1	*CALCR*	*ETS1*	*THBS1*
TMEM37	*TLR4*	*IL1R1*	*PNMA2*
LEPR	*DCN*	*CDKN2C*	*SERPINF1*
ESR1	*LUM*	*TAAR1*	*ALOX5AP*
IL1RN	*TNC*	*CTGF*	*FLT1*
TRH	*CFL1*	*NOS3*	*CD4*
IL1B	*NMT1*	*ENPP1*	*SOD1*
IL1A	*GFAP*	*ESAM*	*GAPDH*
RHO	*NTS*	*CFHR1*	*VWF*
BCL2L11	*SRI*	*FSTL1*	*MMP14*
B3GAT1	*CDH1*	*GSK3B*	*APEX1*
NLRP5	*SLC4A1*	*THY1*	

SHH	*PLA2G2D*	*EPHA1*
OPCML	*CDKN1A*	*FABP7*
IL11	*LCAT*	*ZYX*
NCK2	*PLAU*	*GJA1*
FASTK	*BRCA1*	*ADAM8*
TBPL1	*PTPRB*	*UTF1*
MYLK	*ADAMTS2*	*TIMP2*
IL1R1	*DDX41*	*PLN*
CTGF	*TDGF1*	*EPHB6*
NOS3	*MFAP2*	*PTCH2*
C2orf29	*CDH5*	*BIRC5*
FOXE3	*SMC2*	*PLK3*
HGS	*CCK*	*CXCR5*
CD86	*BAAT*	*ROS1*
EAF2	*CTNNB1*	*HAS1*
EIF5B	*C10orf27*	*MKI67*
NR1I2	*LRRC23*	*CD200*
POMGNT1	*ELN*	*BRAF*
CD80	*PTCH1*	*ADAM12*
THY1	*STX1A*	*KLK13*
HEY2	*ACAN*	*KLK3*
MFRP	*TNMD*	*KLK15*
GAP43	*ACAA1*	*KLK1*
CLCN1	*MMP2*	*IDS*
GJA1	*TAC3*	*PTN*
TIMP2	*CHM*	*PTGDS*

UROD	*BEST1*	*APOA1*
PLN	*PLOD1*	*HTRA1*
BOC	*CNOT8*	*ZBTB16*
PLK3	*PES1*	*ITGB4*
ROS1	*CDKN2A*	*YBX1*
FPR1	*EDN1*	*NOTCH1*
MKI67	*TEK*	*CALD1*
CD200	*USP9X*	*FGFR2*
NPS	*HM13*	*SEC23IP*
CD96	*LY96*	*GRB2*
FYN	*ICAM1*	*NCAM1*
GRIN1	*VIM*	*IL18*
DPP7	*AGT*	*C2*
BACE1	*TNXB*	*MKLN1*
ENTPD2	*WDR36*	*IRF3*
WASF1	*TWIST1*	*ZNF643*
PTGDS	*TIMP1*	*FCGRT*
APOA1	*CFP*	*CD300LF*
EIF2AK3	*PAX6*	*MMP17*
TFG	*MMRN1*	*STX2*
ZBTB16	*CD59*	*ATM*
FOXO3	*WAS*	*MAP3K7*
CALD1	*NPY*	*FOXP1*
BRWD2	*ITCH*	*SOX9*
SEC23IP	*FOS*	*VAMP8*
NCAM1	*CYCS*	*NTF4*

IL18	*CAT*	*LEP*
C2	*TGFB3*	*BAX*
CPA1	*CD44*	*HABP2*
PAEP	*AP003117.1*	*TCF7L2*
POU1F1	*SYP*	*MMP13*
ZMPSTE24	*NOS2*	*FUT1*
OLFM1	*VTN*	*MMP12*
MCHR2	*SMTN*	*MMP3*
RDX	*EGF*	*MMP1*
TSPAN33	*PITX2*	*MMP10*
TRIT1	*BBS9*	*MMP8*
CNTN3	*TGFBI*	*SLC16A6*
ATM	*GRASP*	*MMP7*
HEYL	*SLC6A6*	*BIRC3*
CASP8AP2	*KRT7*	*FEZF2*
ADRB1	*MAZ*	*PRKCA*
SSTR2	*PML*	*CLIP1*
VAMP5	*IL2*	*CAV1*
VAMP8	*FGF2*	*HTRA2*
NTF4	*NUDT6*	*ICAM2*
IMPDH1	*CD14*	*C5AR1*
BAX	*ANXA13*	*GH1*
PDGFD	*MAPK3*	*CYP17A1*
CNR1	*SERPINA4*	*CTSC*
ABO	*TNF*	*SLC17A5*
MMP12	*SDC1*	*PXN*

MTF1	*ITGAL*	*NRCAM*
MMP3	*EDA*	*CHDH*
MMP1	*CYB5R3*	*NOVA2*
WASL	*MAPK8*	*PPP2R4*
MMP8	*IGFBP3*	*DMPK*
FUT2	*TNFRSF9*	*SLC26A3*
ATXN7	*ENO1*	*VASP*
FEZF2	*TGFBR2*	*ITIH4*
MTMR2	*UPP1*	*NOS1*
KNTC1	*SLC18A3*	*PIK3CG*
PRKCA	*TF*	*ACE*
APOH	*ASL*	*MRC2*
COL8A2	*GLB1*	*STAB1*
DIABLO	*EGFR*	*DYSF*
DNASE1L3	*RPS4X*	*RELN*
FLNB	*PDGFRB*	*BCL3*
NUP214	*MMP9*	*PVR*
IMPG1	*ANGPTL7*	*EMD*
FOXP2	*EYA2*	*SERPINH1*
GH1	*MIP*	*FLNA*
HESX1	*SPARC*	*CHUK*
SAE1	*AKT1*	*RPS6KB1*
ASS1	*VCAN*	*IRAK1*
TYR	*MAX*	*PLAUR*
CTSC	*CALCA*	*SDC3*
SLC17A5	*EMP2*	*HPSE2*

PXN	*SDCBP*	*ARD1A*
TOR1B	*IL8*	*MATN1*
CHDH	*AFM*	*TEX101*
NOVA2	*AFP*	*LEFTY2*
DMPK	*FBN1*	*LCN2*
SLC26A4	*ARSB*	*SERPINE1*
F8	*ALB*	*MPO*
OPA3	*COL18A1*	*ACHE*
NOS1	*LYVE1*	*TRPV4*
ZBTB8OS	*MN1*	*POU2F2*
PIK3CG	*IL2RA*	*IL17A*
IKBKG	*FOXC1*	*HYAL1*
ERCC2	*LECT1*	*EPHB4*
G6PD	*TUBB4*	*ACTR2*
ACE	*MLANA*	*ENG*
UBL4A	*SNAP25*	*BGN*
RIMS1	*CTSD*	*CD79A*
APOE	*SERPINB2*	*EPO*
PAX2	*SMN1*	*SEMA3B*
PVR	*EIF5A*	*ETV5*
SCD	*SMN2*	*GALNS*
SERPINH1	*GGT1*	*CD2AP*
CA4	*PLAT*	*TNFRSF21*
FLNA	*BMP2*	*IL18BP*
CHUK	*KIT*	*TGFB1*
ABCC2	*PCNA*	*CYBA*

HCFC1	*ADAL*	*GABRQ*
XRCC1	*PTGER2*	*DNTT*
GRM2	*NID2*	*SFN*
AVPR2	*RB1*	*CTTN*
ZNHIT1	*PRNP*	*RUNX2*
GCLC	*SMARCA2*	*CCND1*
SERPINE1	*ESD*	*VEGFA*
CEACAM1	*AMELX*	*MAPKAP1*
MPO	*BCR*	*TRIM63*
LPO	*FGFR1*	*DAG1*
ACHE	*RIPK4*	*HSPA5*
ZMYND10	*UCHL1*	*MBTPS1*
IL17A	*STAP2*	*XYLT2*
SCPEP1	*MMP26*	*RHOA*
COIL	*CDKN1B*	*CDH13*
ENG	*GP1BA*	*COL1A1*
MMD	*MBP*	*IGF1*
BGN	*MATK*	*EFEMP1*
CD79A	*ITGA2*	*CD40LG*
RPS19P3	*GSR*	*MAF*
EPO	*SOD3*	*FHL1*
PELI1	*BCL2*	*SAGE1*
GALNS	*LDB2*	*NGFR*
TNFRSF21	*IGF2*	*PHB*
CDT1	*CLU*	*BCAR1*
TGFB1	*PROM1*	*GPC3*

GABRQ	*COCH*	*CNR2*
DNTT	*TBX1*	*FAS*
SLC9A1	*GRP*	*SLC25A3*
AXL	*KCNE1*	*CTSF*
CA5A	*THBS1*	*TAC1*
MST1R	*PNMA2*	*GSN*
RUNX2	*NEFM*	*PTEN*
CYP2B7P1	*SERPINF1*	*COL7A1*
FADD	*STC1*	*ITGB3*
FOXC2	*ENO2*	*SNCG*
FOXF1	*ALOX5AP*	*EPHB2*
FGF19	*FLT1*	*NSF*
LMX1B	*CD4*	*COL1A2*
GAL	*CHRNA7*	*RYR1*
TXNRD1	*MRAP*	*TFPI2*
FMR1	*HUNK*	*CDC42*
VEGFA	*SOD1*	*CST6*
MAGEC2	*CHRFAM7A*	*BANF1*
TRIM63	*TJP1*	*CDK6*
REL	*GAPDH*	*HSPG2*
HSPA5	*PRTN3*	*DCN*
AMT	*SETBP1*	*LUM*
RHOA	*VWF*	*PAPPA*
COL1A1	*MMP14*	*MAP3K14*
SGCA	*CDH2*	*XIAP*
IGF1	*RNASE3*	*TNC*

GSTP1	*APEX1*	*KRIT1*
LAMB2	*TGIF1*	*GFAP*
CD40LG	*LPIN2*	*NQO1*
GNPTAB		*SETD2*
CYP26A1		*ITGA2B*
HHEX		*HAS3*
SAGE1		*CDH1*
CLCF1		*CDH3*
RHOD		*CDKN1A*
GPC3		*LTF*
SPTBN1		*CCR5*
LIPA		*ETV7*
CNR2		*AGTR2*
APAF1		*SEMA3A*
FAS		*MAPK14*
GSN		*VCL*
AC009087.2		*PLAU*
HP		*SLCO4A1*
LGALS4		*HGF*
AIFM1		*CD36*
UCN2		*PPARD*
PON1		*MEN1*
SNCG		*LAMA5*
C1QB		*ADRM1*
SASH3		*PTPRB*
TAT		*SPDEF*

COL1A2	*PDLIM7*
OCRL	*IFNG*
CALB2	*MFAP2*
SOCS2	*GRIP1*
CALCR	*ESRRA*
MAPT	*TH1L*
TLR4	*HMGA2*
HSPG2	*LTBP1*
DCN	*STAT3*
LUM	*LEMD3*
KERA	*VEGFB*
XIAP	*CTNNB1*
TNC	*INVS*
KRIT1	*TLR3*
CSPG5	*C10orf27*
KITLG	*MMP15*
GFAP	*PEPD*
DNAJC5	*TGFBR1*
ECE1	*NODAL*
PFTK1	*ACLY*
GLO1	*AIFM2*
CDA	*NKX2-5*
SRI	*RPSA*
CDH1	*BMP7*
SLC4A1	*JUP*
PLA2G2A	*CDK4*

KCNMA1	*GAST*
CDKN1A	*DUSP1*
PSMB10	*AGAP2*
CCR5	*KRT17*
CTRL	*KRT19*
AGTR2	*FGF18*
MAPK14	*FURIN*
TCFL5	*GORASP1*
ACD	*BLM*
VCL	*DOCK2*
CTCF	*CTSL2*
SOS1	*TYMP*
PLAU	*LAT2*
NTSR1	*ANPEP*
AGRP	*VEGFC*
HGF	*LIMK1*
FKBP5	*CCR7*
BRCA1	*HSD17B3*
CYP1B1	*HMMR*
RAMP2	*ARHGAP9*
LYZ	*GLI1*
CPM	*MT3*
CDH4	*ELN*
EIF2AK2	*CLDN4*
TSC22D3	*CEBPB*
HSPB1	*AMFR*

LSR	*NOX1*
IFNG	*PTCH1*
MFAP2	*MYD88*
F12	*CES1*
GRIP1	*ACAN*
HPN	*LRP1*
BAK1	*TNFRSF1B*
LTBP1	*MMP2*
WIF1	*SCRT1*
SMC2	*PALLD*
VEGFB	*HSF1*
BIRC6	*HSD17B6*
POR	*ERBB2*
TBK1	*CNOT8*
GNAS	*CYP21A2*
IGF1R	*NPPB*
CNOT1	*CPE*
GPI	*NPPA*
SRD5A2	*JAG2*
CYP4V2	*CYSLTR1*
XDH	*HAPLN1*
CTNNB1	*CXCL9*
INVS	*EDIL3*
C10orf27	*SOX6*
NODAL	*CXCL10*
ACLY	*RASA1*

BMP7	*CXCL11*
FES	*TFAP2A*
ARSA	*PHYH*
BLM	*CYP19A1*
C19orf2	*RAC1*
AURKA	*IFNB1*
HK1	*THBD*
TAP1	*CXCL13*
SCN5A	*ARG2*
TYMP	*CYBB*
LAT2	*IFNA1*
DDIT3	*FRAS1*
VEGFC	*CST3*
CCR7	*EDN1*
HLA-DQB1	*ANTXR2*
PLIN	*ACTN1*
SBF1	*TEK*
ELN	*MUC16*
HPGD	*BMP3*
CEBPB	*LY75*
HAND2	*MAP2K4*
PTCH1	*PIN1*
SLC25A16	*ABCC6*
SNAI1	*ETV1*
STX1A	*NELL2*
LRP1	*HPSE*

AGER	*DNMT1*
CLCN3	*XYLT1*
CSF3	*SOX4*
MLXIPL	*PRL*
MMP2	*ICAM1*
TNFRSF8	*VIM*
NTRK3	*AC136618.1*
HSF1	*AHR*
CHM	*TIMP3*
MFN2	*AGT*
BEST1	*TNXB*
SGCD	*FABP4*
CNOT8	*PDE4A*
TCAP	*APC*
NPPA	*TWIST1*
FEN1	*RAPGEF3*
NOD2	*MCC*
EGR2	*HMOX1*
MLC1	*EFNB2*
CLCN6	*DNMT3B*
GPHN	*BDNF*
TFAP2A	*ANXA2*
DCT	*SPARCL1*
PES1	*MAPRE1*
PDE7A	*ZNF410*
CYP19A1	*DSPP*

NUCB2	*TIMP1*
IFNB1	*DMP1*
ARG2	*IBSP*
IFNA2	*MEPE*
IFNA1	*COL4A1*
EDN1	*SPP1*
ADAMTS10	*COL2A1*
ANTXR2	*HIST1H1C*
VSX1	*DNM2*
SAA2	*COL4A2*
SAA1	*IL6*
BMP3	*MMP16*
USP9X	*LOX*
CD83	*SMARCA4*
CAST	*CCL19*
MAP2K4	*LTBP2*
NYX	*MMRN1*
LDHA	*PGF*
NINL	*WAS*
OLFM2	*WNT1*
ABCC6	*FBN2*
PTPN5	*FOS*
ANGPTL6	*DHH*
HM13	*CYCS*
ID1	*CAT*
BCL2L1	*ELAVL3*

PRL	*TGFB3*
ICAM1	*CD44*
VIM	*CCNE2*
ICAM4	*VHL*
AC136618.1	*LAMP1*
SMG1	*PRPH*
HEY1	*PIM2*
AHR	*IL3*
TIMP3	*DPP8*
AGT	*CSF2*
WDR36	*SDC2*
APC	*PPP3CA*
SNX16	*MMP24*
TWIST1	*TRAF6*
ERCC5	*RAG1*
HMOX1	*MATN2*
EFNB2	*NFKB1*
CA1	*PPP3R1*
CA2	*PRKCB*
BDNF	*IL5*
ARAF	*LGALS1*
TIMP1	*AGRN*
COL4A1	*IL13*
SPP1	*IL4*
COL2A1	*EXT2*
HFE	*RECK*

IL6	*MAP2K1*
SENP1	*MAP2K3*
PAX6	*TIMP4*
VSX2	*PPARG*
ABCD4	*SMAD6*
LOX	*NOS2*
DECR1	*SMAD3*
WT1	*SEL1L*
TPM1	*RAF1*
CCL19	*LEF1*
CCL21	*HLA-E*
LTBP2	*VTN*
GPNMB	*ZEB1*
CA12	*CALR*
SNCA	*TGOLN2*
LDLR	*SMTN*
MMRN1	*SPAG5*
CD59	*GLCE*
PDLIM5	*CTHRC1*
PGF	*EGF*
WAS	*WNT7A*
PVALB	*TGFBI*
SLC12A2	*LRP12*
FBN2	*TBX20*
EPOR	*ANGPT1*
FOS	*ACVRL1*

F7	*NPTXR*
CYCS	*KRT7*
CAT	*PDGFB*
HINT1	*ADAM17*
IL2RB	*EBAG9*
TGFB3	*DDR1*
RAC2	*CD19*
RASL12	*FBLN5*
MAPK7	*EGR1*
CD44	*ODC1*
VHL	*KRT8P9*
LAMP1	*SRC*
CA9	*ANXA5*
SLC1A2	*EXT1*
CSF2	*GIT1*
GSTT1	*TNFRSF11B*
NGB	*NOV*
GSTZ1	*ENPP2*
NFKB1	*TGM2*
IRF1	*LOXL1*
IL5	*COL14A1*
MOG	*HBEGF*
RAD50	*EP300*
LGALS1	*IL2*
AQP2	*RET*
ALKBH1	*FGF2*

IL4	*NUDT6*
HLA-A	*HAS2*
SYP	*CXCL12*
PRDX2	*MYCN*
BAMBI	*AEBP1*
SMAD6	*MAPK3*
NOS2	*CSK*
SMAD3	*SERPINA1*
RAF1	*ANXA1*
HADH	*SP1*
CLCN5	*MYL7*
HLA-E	*KAT2B*
VTN	*MSN*
ZEB1	*MATN3*
CALR	*TNF*
CLN6	*MYC*
SMTN	*SDC1*
SPAG5	*ITGAL*
EGF	*CCL2*
ALLC	*APOB*
RIMS2	*CCL7*
NEUROG1	*EDA*
PITX2	*CCL11*
BBS9	*CCL8*
TGFBI	*PPIA*
SLC6A6	*CSPG4*

NR2E3	*FGF1*
KRT7	*MIF*
UGT8	*SERPING1*
PDGFB	*WISP1*
FLOT1	*ITGA5*
LPHN1	*MAPK8*
PDE5A	*FOXO4*
EGR1	*IGFBP1*
KRT3	*IGFBP3*
HSPA9	*VAMP3*
GRAP2	*PARK7*
PTGER1	*MMP28*
KRT8P9	*PPP1R1A*
ANXA5	*EDNRA*
CTNNBL1	*SDC4*
KRT18P19	*ADCY3*
ROCK2	*CCL5*
TGM2	*TGFBR2*
LOXL1	*NONO*
NRG2	*CCL3*
COL14A1	*PTK2*
IL2	*CCL4*
RET	*POMC*
FGF2	*TF*
CDIPT	*BST2*
NUDT6	*NCOA4*

TRPM3	*ADRB2*
INTU	*HSP90AA2*
NOTCH3	*CTSH*
CYP1A1	*MMP19*
ANXA13	*ITGAM*
CYP1A2	*CDK2*
MAPK3	*GLB1*
SERPINA6	*CXCR3*
SERPINA1	*MS4A2*
MTSS1	*ERBB3*
SERPINA3	*EGFR*
CYP4F3	*RPS4X*
HEPH	*FBLN1*
LTA	*CTSL1*
CD2BP2	*CSF1R*
TNF	*CCRK*
CYP2D6	*PDGFRB*
ITGAL	*MMP9*
RAB8A	*CD40*
ACOT7	*PPARA*
CBX5	*CKB*
EFNB1	*JUND*
GYPB	*MAPRE3*
FGF1	*SYK*
SMTNL1	*ROR2*
YWHAB	*SPTLC1*

ADCY1	*RXFP1*
MAPK8	*COMP*
WARS	*MIP*
TNFRSF9	*SPARC*
SLPI	*AKT1*
POU4F2	*ADCY7*
PPP1R1A	*OGN*
EDNRA	*VCAN*
GJB1	*MAX*
ADCY3	*DMD*
ENO1	*CALCA*
TGFBR2	*FSCN1*
UPP1	*DSP*
CHAT	*FGF7*
EIF2C2	*EDNRB*
SLC18A3	*CIITA*
POMC	*OSM*
NTRK2	*LIF*
H6PD	*COPS2*
TF	*ACTB*
GLYAT	*EPGN*
GDF11	*ESR2*
ADRB2	*PTH*
HSP90AA2	*CXCL5*
WDR20	*PPBP*
SLURP1	*PF4*

SILV	*IL8*
HSPA1A	*RETN*
HSPA1B	*F13A1*
CCR4	*AFM*
BCL2A1	*COL6A2*
GLB1	*PARVA*
NEU1	*FBN1*
EGFR	*ALB*
GAS1	*FREM1*
RPS4X	*PHEX*
PDE6A	*F2RL1*
AL117209.1	*F2R*
CSF1R	*RRBP1*
MMP9	*PTHLH*
APOBEC3G	*COL18A1*
CD40	*DSTN*
ANGPTL7	*GNA12*
PLEC1	*EWSR1*
COMP	*HIF1A*
MLH1	*HMGCR*
CPNE1	*LYVE1*
RBM12	*ITGB2*
BRD1	*SNAI2*
MIP	*ADM*
SPARC	*SSPN*
AKT1	*STATH*

ADCY7	*CSN2*
MTHFR	*CEBPD*
OGN	*TP53*
VCAN	*LECT1*
OPTN	*CREBBP*
MAX	*TUBB4*
XRCC4	*JAG1*
CALCA	*TMPRSS11D*
BMP6	*CTSD*
MAP2K7	*SERPINB2*
CDC123	*RLN2*
POU4F1	*OTX2*
FGF7	*JAK2*
EDNRB	*IGFBP7*
ACTB	*IKBKB*
MTHFD1	*HOPX*
ESR2	*MMP25*
PTH	*PLAT*
CXCL2	*BMP2*
SDCBP	*PDGFA*
FCER2	*NRTN*
IL8	*LGALS3*
USP6NL	*CLDN7*
TEAD1	*GRPR*
NF2	*OCLN*
PER1	*KLF6*

PSIP1	*TAF9*
FBN1	*KDR*
OVOL2	*AC145132.3*
NEFH	*FIGF*
RP1	*KIT*
TMTC1	*CBS*
ALB	*BMP4*
PHEX	*SFRP1*
COL18A1	*ILK*
PPL	*IDO1*
OTOR	*PCNA*
HIF1A	*RASSF2*
SLC4A4	*ADRA1D*
SIX1	*MMP11*
TYRP1	*ADAM9*
ADM	*MGP*
HMOX2	*ATL1*
VAV1	*FGFR1*
SBF2	*MLST8*
MN1	*MAPK1*
IL2RA	*MMP26*
B2M	*BACE2*
OLFM4	*CDKN1B*
SULT1E1	*MAP3K1*
TP53	*EPSTI1*
ADCY9	*TNFSF11*

FOXC1	*GP1BA*
FOXQ1	*IL6ST*
SHBG	*MBP*
TNFSF14	*ESM1*
CREBBP	*FST*
BTBD3	*ITGA2*
CARTPT	*ITGA1*
CD68	*ETS2*
JAG1	*FGF10*
AIRE	*OLR1*
TMPRSS11D	*NFKBIA*
PDCD1LG2	*DUSP4*
CTSD	*POSTN*
CD274	*SOD3*
LPHN3	*PPARGC1A*
NHS	*CD81*
PLCB4	*A2M*
IKBKB	*SERPINB5*
AKR1C4	*BCL2*
AKR1C3	*C4A*
EIF5A	*TNFRSF11A*
GGT1	*INS*
PLAT	*IGF2*
BMP2	*CLU*
PDGFA	*PROM1*
LGALS3	*TPSAB1*

AKR1C2	*RUNX1*
GLIS3	*PTK2B*
AKR1C1	*RCAN1*
ACE2	*PRKD1*
PIR	*SLC5A3*
KIT	*GZMB*
CBS	*THBS1*
BMP4	*PNMA2*
SFRP1	*C1R*
ILK	*C1S*
FANCB	*STUB1*
PCNA	*SERPINF1*
PTGER2	*PTPN6*
HBB	*SERPINF2*
RB1	*ALOX5AP*
PRNP	*HMGB1*
ADRA1D	*LOXL2*
SMPD1	*SMAD4*
TFF1	*FLT1*
GTPBP4	*CD151*
HTR2A	*CD4*
HTR1A	*YWHAE*
AC055839.1	*ZNF384*
BCR	*LIPG*
MGP	*MSX1*
SAV1	*DYM*

ERCC8	*SMAD7*
EMP1	*SMAD2*
TPT1	*BMP1*
MAPK1	*JPH4*
TSC22D1	*TRIO*
BACE2	*LRPAP1*
CDKN1B	*SOD1*
SLC4A11	*MYH7*
ARSD	*C16orf35*
ADRB3	*TJP1*
TRIM21	*GAPDH*
UBE2K	*IFITM1*
GP1BA	*ELANE*
TSC2	*PRTN3*
STK19P	*FGF9*
MBP	*LPL*
ESM1	*ADAMTS5*
CNDP1	*FSTL3*
FOXO1	*JUB*
TBC1D1	*ADAMTS1*
SSTR1	*VWF*
ISL1	*APP*
FGF10	*NTF3*
CCL28	*BSG*
GSR	*BGLAP*
NFKBIA	*MMP14*

POSTN	*FGF23*
PTGER4	*NRIP1*
SERPINB3	*MEP1B*
A2M	*CDH2*
CTNS	*CTSB*
BCL2	*GATA4*
PBK	*APEX1*
C4A	*GATA6*
LDB2	*ANGPT2*
TNFRSF11A	*ROCK1*
COMT	*TWSG1*
INS	
IGF2	
GDNF	
CLU	
COCH	
HLA-DRB3	
EPHX2	
ADCY10	
SLC1A3	
MSLN	
PEX5	
THBS1	
PNMA2	
NEFL	
NEFM	

C1S
STUB1
SERPINF1
SERPINF2
ENO2
ALOX5AP
LOXL2
C4B
NPR3
TNFRSF10A
BID
FLT1
TNFRSF10B
PDX1
CD4
YWHAE
SNPH
NRL
GREM1
SMAD7
SMAD2
BMP1
MRAP
LRPAP1
CENPJ
SOD1

C16orf35
MPG
HTT
GAPDH
SGCG
SETBP1
TNFRSF1A
PSMB5
VWF
AQP1
APP
TERT
NTF3
BSG
GJB2
MMP14
SLC7A7
BTG3
IDUA
FOXM1
TTR
FKBP4
PDE6B
AQP4
APEX1
MIB1

ROCK1
ZFP161
DLGAP1
TGIF1
AC217779.1

Table S2. Summary of biological process gene set enrichment analysis of text mining genes.

Process	Genes in query set	Total genes in genome	Corrected hypergeometric P value	Genes
Extracellular matrix organization	31	257	3.47E-27	*CDH1,CD44,VWF,VTN,VCAM1,TNF,THBS1,TGFBI,SPARC,BGN,SERPINE1,NID1,MMP14,MMP9,MMP3,MMP2,MMP1,MFAP2,LUM,ITGAL,ICAM1,TNC,FN1,FGF2,FBN1,ELN,COL18A1,DCN,VCAN,COL1A2,COL1A1*
Platelet degranulation	20	125	2.44E-19	*CLU,MMRN1,VWF,VEGFA,TIMP1,THBS1,TGFB3,TGFB2,TGFB1,TF,SPARC,SOD1,SERPINE1,KNG1,IGF2,IGF1,APOA1,FN1,ALB,EGF*
Cytokine-mediated signaling pathway	25	290	3.99E-18	*CDKN1A,CD4,VIM,VEGFA,VCAM1,TWIST1,TNF,TIMP1,TGFB1,BCL2,NOS2,MMP9,MMP3,MMP2,MMP1,KIT,IL8,IL2,ICAM1,FOS,FN1,FGF2,AKT1,F3,COL1A2*
Response to drug	22	305	3.82E-14	*CDKN1B,CDKN1A,CDH1,CAT,TIMP2,THBS1,TGFBR2,TGFB2,SST,SOD1,BCL2,REN,PTCH1,APOA1,ICAM1,APEX1,FOS,ENG,EDN1,COL18A1,CTNNB1,COL1A1*
Positive regulation of cell population Proliferation	27	557	1.45E-13	*CDKN1B,CNOT8,VEGFA,TIMP1,THBS1,TGFBR2,TGFB3,TGFB2,TGFB1,SHH,RPS4X,BCL2,KIT,IL2,IGF2,IGF1,TNC,FN1,FLT1,FGF2,AKT1,AGT,EGFR,EGF,EDN1,COL18A1,CTNNB1*
Response to hypoxia	17	174	7.16E-13	*CDKN1B,CAT,VEGFA,THBS1,TGFBR2,TGFB3,TGFB2,BMP2,PLAU,PLAT,NOS2,NOS1,MMP14,MMP2,ICAM1,ENG,EDN1*
Wound healing	14	103	1.99E-12	*TIMP1,TGFBR2,TGFB3,TGFB2,SPARC,IGF1,TNC,GSN,FN1,FGF2,ENG,EGFR,DCN,COL1A1*
Regulation of cell population Proliferation	18	222	2.05E-12	*CLU,CDKN1B,TNF,TGFBR2,TGFB3,TGFB2,TGFB1,SPARC,SHH,PTCH1,PLAU,NOS2,KIT,TNC,ENG,AGT,EGFR,CTNNB1*
Negative regulation of gene expression	20	310	4.16E-12	*CDKN1A,CD34,VEGFA,TNF,TGFB2,TGFB1,BMP2,SHH,SERPINF1,NOS2,MAX,IL8,IGF1,FGF2,AKT1,ENG,AGT,EDN1,ACE,CTNNB1*
Positive regulation of gene expression	24	498	4.56E-12	*CLU,CD34,VIM,VEGFA,TWIST1,TNF,TGFB1,BMP2,SHH,MAPK8,MAPK3,NOS3,KIT,IL8,IGF1,TNC,GSN,GJA1,FN1,AKT1,F3,ENG,EGF,CTNNB1*
Positive regulation of cell migration	18	255	1.66E-11	*VEGFA,THBS1,TGFB1,BMP2,SOD2,PLAU,MMP14,MMP9,KIT,IGF1,HSPA5,FLT1,F3,EGFR,EGF,EDN1,COL18A1,COL1A1*
Aging	15	178	1.57E-10	*CAT,CALCA,TIMP2,TIMP1,TGFBR2,TGFB3,SOD1,MAPK3,SERPINF1,APEX1,GSN,FOS,AKT1,AGT,DCN*
Cellular protein metabolic process	16	226	3.55E-10	*CALCA,TIMP1,TGFBI,TF,MMP2,MMP1,KNG1,IGF2,IGF1,APOA1,TNC,GSN,FN1,ALB,FBN1,VCAN*
Regulation of blood pressure	10	63	1.50E-09	*CD34,CALCA,SOD2,SOD1,REN,NOS3,AGT,EDN1,ACE,COL1A2*
Positive regulation of MAP kinase activity	10	63	1.50E-09	*VEGFA,TNF,TGFB1,KIT,GH1,FLT1,FGF2,EGFR,EGF,EDN1*
Response to estradiol	12	111	1.51E-09	*CDKN1B,CD4,CAT,TGFB1,PTCH1,PCNA,GJA1,GH1,AGT,EGFR,CTNNB1,COL1A1*
Extracellular matrix disassembly	10	67	2.69E-09	*CD44,TIMP2,TIMP1,MMP14,MMP9,MMP3,MMP2,MMP1,GSN,ENG*
Positive regulation of peptidyl-tyrosine phosphorylation	11	92	2.93E-09	*CD44,CD4,VTN,VEGFA,TGFB1,IGF2,IGF1,ICAM1,GH1,AGT,EGF*
Cellular response to growth factor stimulus	10	69	3.26E-09	*CAT,TWIST1,THBS1,TGFBR2,SPARC,BMP2,NOS1,AKT1,EGFR,CTNNB1*
Kidney development	12	122	3.28E-09	*CAT,VEGFA,TGFB2,SHH,BCL2,REN,SERPINF1,FBN1,AGT,ACE,DCN,CTNNB1*
Cell adhesion	23	655	5.32E-09	*MMRN1,CDH1,CD44,CD34,CD4,VWF,VTN,VCAM1,THBS1,TGFBI,PVR,NID1,ACHE,ITGAL,IL2,ICAM1,TNC,GP1BA,FN1,ENG,COL18A1,CTNNB1,VCAN*

Heart development	15	248	1.01E-08	*CDKN1B,CDKN1A,TGFBR2,TGFB2,TGFB1,SPARC,BMP2,SHH,PCNA,GJA1,FN1,FBN1,ENG,EDN1,CTNNB1*
Cellular response to cadmium ion	8	38	1.19E-08	*SOD1,MAPK8,MAPK3,MMP9,GSN,FOS,AKT1,EGFR*
Response to mechanical stimulus	9	65	4.01E-08	*THBS1,TGFBR2,PTCH1,MAPK8,MMP14,TNC,FOS,DCN,COL1A1*
Positive regulation of angiogenesis	12	156	4.35E-08	*CD34,VEGFA,TWIST1,THBS1,TGFBR2,SERPINE1,NOS3,IL8,FLT1,FGF2,F3,ENG*
Skeletal system development	12	155	4.36E-08	*TGFB2,BMP2,MMP14,MMP9,IGF2,IGF1,FBN1,EDN1,CTNNB1,VCAN,COL1A2,COL1A1*
Negative regulation of apoptotic process	20	547	4.51E-08	*CDKN1B,CDKN1A,CD44,CAT,VEGFA,TWIST1,TIMP1,THBS1,SOD1,SHH,BCL2,MAPK8,MMP9,IL2,IGF1,HSPA5,ALB,AKT1,EGFR,CTNNB1*
Positive regulation of epithelial to mesenchymal transition	8	46	5.01E-08	*TWIST1,TGFBR2,TGFB3,TGFB2,TGFB1,BMP2,CTNNB1,COL1A1*
Positive regulation of cell division	8	48	6.69E-08	*CAT,VEGFA,TGFB3,TGFB2,TGFB1,SHH,IGF2,FGF2*
Activation of MAPK activity	11	128	6.74E-08	*TNF,THBS1,TGFB3,BMP2,SOD1,MAPK3,KIT,IGF1,FGF2,AGT,EGF*

Table S3. Summary of Kyoto Encyclopedia of Genes and Genomes (KEGG) pathway gene set enrichment analysis.

Process	Genes in query set	Total genes in genome	Corrected hypergeometric P value	Genes
AGE-RAGE signaling pathway in diabetic complications	23	100	1.04E-22	*CDKN1B,VEGFA,VCAM1,TNF,TGFBR2,TGFB3,TGFB2,TGFB1,BCL2,MAPK8,MAPK3,SERPINE1,NOS3,MMP2,IL8,ICAM1,FN1,AKT1,F3,AGT,EDN1,COL1A2,COL1A1*
Proteoglycans in cancer	26	205	3.04E-19	*CDKN1A,CD44,VTN,VEGFA,TWIST1,TNF,THBS1,TGFB2,TGFB1,SHH,PTCH1,MAPK3,PLAU,MMP9,MMP2,LUM,IGF2,IGF1,FN1,FGF2,AKT1,EGFR,DCN,CTNNB1,COL1A2,COL1A1*
Pathways in cancer	34	531	1.88E-16	*CDKN1B,CDKN1A,CDH1,VEGFA,TGFBR2,TGFB3,TGFB2,TGFB1,BMP2,SHH,BCL2,PTCH1,MAPK8,MAPK3,NOS2,MMP9,MMP2,MMP1,MAX,KNG1,KIT,IL8,IL2,IGF2,IGF1,FOS,FN1,FGF2,AKT1,AGT,EGFR,EGF,EDN1,CTNNB1*
HIF-1 signaling pathway	16	109	1.17E-12	*CDKN1B,CDKN1A,VEGFA,TIMP1,TF,BCL2,MAPK3,SERPINE1,NOS3,NOS2,IGF1,FLT1,AKT1,EGFR,EGF,EDN1*
Relaxin signaling pathway	17	129	1.34E-12	*VEGFA,TGFBR2,TGFB1,MAPK8,MAPK3,NOS3,NOS2,NOS1,MMP9,MMP2,MMP1,FOS,AKT1,EGFR,EDN1,COL1A2,COL1A1*
Chagas disease	15	102	6.57E-12	*TNF,TGFBR2,TGFB3,TGFB2,TGFB1,MAPK8,MAPK3,SERPINE1,NOS2,KNG1,IL8,IL2,FOS,AKT1,ACE*
Bladder cancer	11	41	1.05E-11	*CDKN1A,CDH1,VEGFA,THBS1,MAPK3,MMP9,MMP2,MMP1,IL8,EGFR,EGF*
PI3K-Akt signaling pathway	23	354	5.42E-11	*CDKN1B,CDKN1A,VWF,VTN,VEGFA,THBS1,BCL2,MAPK3,NOS3,KIT,IL2,IGF2,IGF1,TNC,GH1,FN1,FLT1,FGF2,AKT1,EGFR,EGF,COL1A2,COL1A1*
Colorectal cancer	13	86	1.41E-10	*CDKN1A,TGFBR2,TGFB3,TGFB2,TGFB1,BCL2,MAPK8,MAPK3,FOS,AKT1,EGFR,EGF,CTNNB1*
Prostate cancer	13	97	6.18E-10	*CDKN1B,CDKN1A,BCL2,MAPK3,PLAU,PLAT,MMP9,MMP3,IGF1,AKT1,EGFR,EGF,CTNNB1*
Focal adhesion	17	201	7.47E-10	*VWF,VTN,VEGFA,THBS1,BCL2,MAPK8,MAPK3,IGF1,TNC,FN1,FLT1,AKT1,EGFR,EGF,CTNNB1,COL1A2,COL1A1*
Gastric cancer	15	149	9.16E-10	*CDKN1B,CDKN1A,CDH1,TGFBR2,TGFB3,TGFB2,TGFB1,SHH,BCL2,MAPK3,FGF2,AKT1,EGFR,EGF,CTNNB1*
FoxO signaling pathway	14	131	1.761E-09	*CDKN1B,CDKN1A,CAT,TGFBR2,TGFB3,TGFB2,TGFB1,SOD2,MAPK8,MAPK3,IGF1,AKT1,EGFR,EGF*
Fluid shear stress and atherosclerosis	14	139	3.65E-09	*VEGFA,VCAM1,TNF,BCL2,MAPK8,PLAT,NOS3,MMP9,MMP2,ICAM1,FOS,AKT1,EDN1,CTNNB1*
Rheumatoid arthritis	12	93	3.967E-09	*VEGFA,TNF,TGFB3,TGFB2,TGFB1,MMP3,MMP1,ITGAL,IL8,ICAM1,FOS,FLT1*
Pancreatic cancer	11	76	6.112E-09	*CDKN1A,VEGFA,TGFBR2,TGFB3,TGFB2,TGFB1,MAPK8,MAPK3,AKT1,EGFR,EGF*
Hepatitis B	14	162	2.3154E-08	*CDKN1A,TNF,TGFBR2,TGFB3,TGFB2,TGFB1,BCL2,MAPK8,MAPK3,PCNA,MMP9,IL8,FOS,AKT1*
MAPK signaling pathway	18	294	2.3289E-08	*VEGFA,TNF,TGFBR2,TGFB3,TGFB2,TGFB1,MAPK8,MAPK3,MAX,KIT,IGF2,IGF1,FOS,FLT1,FGF2,AKT1,EGFR,EGF*
Malaria	9	50	2.9003E-08	*VCAM1,TNF,THBS1,TGFB3,TGFB2,TGFB1,ITGAL,IL8,ICAM1*
Endocrine resistance	11	98	7.869E-08	*CDKN1B,CDKN1A,BCL2,MAPK8,MAPK3,MMP9,MMP2,IGF1,FOS,AKT1,EGFR*
